# Carbon Catabolite Repression Governs Diverse Physiological Processes and Development in Aspergillus nidulans

**DOI:** 10.1128/mbio.03734-21

**Published:** 2022-02-15

**Authors:** Yingying Chen, Liguo Dong, Md Ashiqul Alam, Lakhansing Pardeshi, Zhengqiang Miao, Fang Wang, Kaeling Tan, Michael J. Hynes, Joan M. Kelly, Koon Ho Wong

**Affiliations:** a Faculty of Health Sciences, University of Macau, Macau SAR, China; b Department of Genetics and Evolution, School of Biological Science, The University of Adelaide, Adelaide, Australia; c Genomics and Bioinformatics Core, Faculty of Health Sciences, University of Macau, Macau SAR, China; d Department of Genetics, The University of Melbourne, Victoria, Australia; e Institute of Translational Medicine, Faculty of Health Sciences, University of Macau, Macau SAR, China; f MoE Frontiers Science Center for Precision Oncology, University of Macau, Macau SAR, China; University of Melbourne

**Keywords:** carbon catabolite repression, carbon metabolism, fungal physiology, gene regulation, transcription factor

## Abstract

Carbon catabolite repression (CCR) is a common phenomenon of microorganisms that enable efficient utilization of carbon nutrients, critical for the fitness of microorganisms in the wild and for pathogenic species to cause infection. In most filamentous fungal species, the conserved transcription factor CreA/Cre1 mediates CCR. Previous studies demonstrated a primary function for CreA/Cre1 in carbon metabolism; however, the phenotype of *creA*/*cre1* mutants indicated broader roles. The global function and regulatory mechanism of this wide-domain transcription factor has remained elusive. Here, we applied two powerful genomics methods (transcriptome sequencing and chromatin immunoprecipitation sequencing) to delineate the direct and indirect roles of Aspergillus nidulans CreA across diverse physiological processes, including secondary metabolism, iron homeostasis, oxidative stress response, development, N-glycan biosynthesis, unfolded protein response, and nutrient and ion transport. The results indicate intricate connections between the regulation of carbon metabolism and diverse cellular functions. Moreover, our work also provides key mechanistic insights into CreA regulation and identifies CreA as a master regulator controlling many transcription factors of different regulatory networks. The discoveries for this highly conserved transcriptional regulator in a model fungus have important implications for CCR in related pathogenic and industrial species.

## INTRODUCTION

Fungi can break down and metabolize a wide range of carbon sources for growth. Efficient and versatile utilization of nutrients is fundamental for saprophytic species to thrive in the wild and for pathogenic species to infect a host. Fungi have evolved elaborate regulatory mechanisms to control their metabolism according to substrate quality and availability. When a mixture of carbon substrates is available, fungi preferentially utilize favorable carbon sources over alternative ones.

Carbon catabolite repression (CCR) is the key transcriptional regulation mechanism underlying the selective utilization of carbon sources (1, 2). CCR has been extensively studied in the yeast Saccharomyces cerevisiae and the filamentous fungus Aspergillus nidulans. In S. cerevisiae, the DNA-binding repressor Mig1 and, to a lesser extent, its paralog Mig2 are the main regulatory proteins mediating CCR (3–6), while CreA is the key regulator of CCR in A. nidulans (7–9). Although both *MIG1* and *creA* genes encode C_2_H_2_ zinc finger proteins recognizing a similar DNA binding consensus sequence (SYGGRG) (7, 10–13), there are functional differences between the two transcriptional regulators (14, 15).

In A. nidulans, CreA controls carbon metabolism through both direct and indirect mechanisms. Direct CreA binding to the promoter of its target genes can control promoter accessibility through affecting nucleosome positioning (16) and/or compete with pathway-specific transcription factors for DNA binding (10, 17–19). CreA can also indirectly regulate genes through controlling expression of pathway-specific transcription factors that activate distinct sets of metabolic genes (10, 12, 13, 17, 18, 20–26). Genes in some metabolic pathways are subject to both direct and indirect CreA controls (10, 22), presumably to ensure their tight repression.

CreA function is highly regulated. At the transcriptional level, the *creA* gene is differentially controlled by carbon sources and subjected to autogenous regulation (i.e., by CCR) (27, 28). The autogenous control may serve as a feedback mechanism to fine-tune its expression, although this control does not appear to be necessary for CreA repression, as demonstrated in a *creA* overexpression strain (29). At the protein level, CreA is localized in the nucleus under conditions that lead to strong repression (29, 30), but its subcellular localization under conditions that are not strongly repressing, or indeed starving, is controversial. In one study, it was found that nuclear localization of overexpressed CreA-green fluorescent protein (GFP) is not affected by CCR conditions (29). In other studies, CreA-GFP (expressed from its endogenous promoter) subcellular localization was regulated according to CCR conditions (30–32). Moreover, CreA protein is also subject to degradation (27, 29–31). The roles of cellular compartmentalization and degradation are unclear, but studies have shown that both repression and derepression can occur when CreA is present in the nucleus (29), suggesting that additional posttranslational control is required for regulating CreA activity (27, 29). Phosphorylation has been implicated as the main control of CreA activity (33–35), and potential kinases and phosphorylation sites for CreA regulation have been identified (32, 33, 35, 36).

Previous studies have established CreA (and its orthologues in other fungi) mainly as a repressor of carbon metabolic genes (7, 10, 13, 14, 17, 19, 22, 37–43). Recently, studies in various fungal species indicate additional CreA functions beyond carbon metabolism. For example, the *creA*Δ mutants of Aspergillus flavus (44), Magnaporthe oryzae (45), and Beauveria bassiana (46) are affected in development and virulence, while repression of genes with functions in nitrogen uptake, development, chromatin remodeling, and the mediator complex depends on a functional CreA in Trichoderma reesei (41). In Aspergillus fumigatus, the *creAΔ* mutant impacts growth, fitness, and virulence (47). In all these cases, it is not known whether these noncarbon processes are directly controlled by the respective CreA orthologues or are indirect consequences of defective carbon metabolism, growth, and/or stress response.

In A. nidulans, the severe growth defect of some *creA* mutants suggests pleiotropy (28, 48). However, the full extent of genome-wide functions of this important transcriptional regulator is unknown. Here, we combine two powerful genomic techniques, RNA-seq (transcriptome sequencing) and ChIP-seq (chromatin immunoprecipitation followed by sequencing), to study CreA regulation and function at the genome-wide level. The overall results not only delineate the direct and indirect roles of CreA under different conditions but also identify the global regulatory network of CreA. This work also reveals that, through modulating the hierarchy of different regulatory networks, CreA is a master regulator of diverse processes in addition to carbon metabolism.

## RESULTS

### Generation and phenotypic characterization of a *creA*Δ mutant.

Many *creA* mutants have been isolated and shown to have different growth phenotypes (48, 49). We generated a deletion mutant removing the entire *creA* coding region and compared its growth phenotype on different carbon sources with the *creA99* mutant, which lacks the entire *creA*-coding region except for the first 46 amino acids and is commonly considered a null mutant (28). The new *creA*Δ mutants showed the same morphological defects as the *creA99* mutant, with the growth of the *creA*Δ mutant being slightly weaker than that of *creA99* under all conditions tested (see Fig. S1A in the supplemental material). This subtle difference may be due to an effect of the N-terminal peptide or, more likely, different genetic backgrounds between the strains. Nevertheless, both the *creA99* and new *creA*Δ mutants have the carbon derepression phenotype, as indicated by increased sensitivity to a toxic analogue of ethanol (allyl alcohol) (Fig. S1B) and milk clearing around the colony (Fig. S1C) when growing on solid media with 1% glucose. Therefore, the newly generated *creA*Δ strain has the expected phenotype.

10.1128/mbio.03734-21.1 FIG S1 Growth phenotype of the wild-type, *creAΔ* and *creA99* strains under different carbon conditions. (A) Plate tests of the wild-type, *creAΔ*, and *creA99* strains grown on solid ANM medium containing the indicated carbon compound as the sole carbon source at 37°C for 3 days. (B) Plate tests for the wild-type, *creAΔ*, and *creA99* strains on solid ANM medium containing 1% glucose and different alcohol concentrations at 37°C for 3 days. (C) Plate tests for the wild-type, *creAΔ*, and *creA99* strains in solid ANM medium containing 1% glucose and 1% skim milk at 37°C for 3 days. The phenotype of the wild type after 1 day is presented for reference. Download FIG S1, PDF file, 2.0 MB. Copyright © 2022 Chen et al. 2022 Chen et al. https://creativecommons.org/licenses/by/4.0/ This content is distributed under the terms of the Creative Commons Attribution 4.0 International license.

### RNA-seq analysis identified more than a thousand genes under CreA control during growth under CCR conditions.

**FIG 1:**
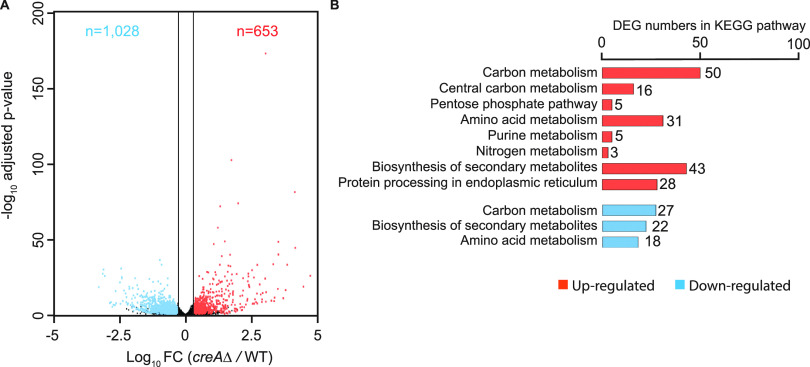
RNA-seq analysis reveals the effect of *creA* deletion on more than a thousand genes under CCR conditions. (A) A volcano plot displaying gene expression changes between the wild type (WT) and the *creAΔ* mutant. The *x* axis shows log_10_ fold change (FC) between the WT and the *creAΔ* mutant, while the *y* axis presents −log_10_ adjusted *P* values. The significantly up- and downregulated genes are shown in red and blue, respectively. (B) A bar plot presenting the representative KEGG pathways for the DEGs between the WT and the *creAΔ* mutant. KEGG pathways of upregulated and downregulated genes are colored in red and blue, respectively.

To gain insight into the genome-wide role of CreA, RNA-seq was performed on the wild type and the *creA*Δ mutant under CCR conditions (1% glucose). Compared to the wild type, 653 and 1,028 genes were up- and downregulated in the mutant, respectively (Fig. 1A; also see Data Set S1 at https://figshare.com/s/9f324a2370691f209579). Consistent with its well-established role, the upregulated genes are highly enriched with functions in carbon metabolism (Fig. 1B and Data Set S2 available at https://figshare.com/s/9f324a2370691f209579). A significant number of genes from the pentose phosphate pathway (*n* = 5), amino acid (*n* = 31), purine (*n* = 5), and nitrogen (*n* = 3) metabolism, biosynthesis of secondary metabolites (*n* = 43), and protein processing in endoplasmic reticulum (*n* = 28) were also upregulated in the *creA*Δ mutant. On the other hand, only a few pathways (e.g., carbon, amino acid, and secondary metabolism) are enriched among downregulated genes (Fig. 1B and Data Set S2). Overall, the RNA-seq result revealed ∼15% of the A. nidulans genome is affected by the loss of CreA in glucose medium.

### CreA binds to about two thousand genomic locations under CCR conditions.

Derepression of many genes, including those repressed by CreA, requires transcriptional activation by pathway-specific transcription factors whose function often depends on the presence of low-molecular-weight inducer molecules (50). As such, expression of CreA targets may not significantly increase in the *creA*Δ mutant if the relevant pathway-specific transcription factor is not expressed or its corresponding inducer is absent under the growth condition analyzed. Consequently, these genes would not be detected by measuring changes in mRNA levels. To identify these, we mapped CreA occupancies in the genome using ChIP-seq. Two different epitope-tagged versions of CreA (CreA^HA^ and CreA^GFP^) were used to avoid antibody and epitope biases. Since the N terminus of CreA is subjected to posttranslational processing (29) and to minimize effects on the DNA binding domain located near the N-terminal region, both tags were introduced to the C terminus of CreA. The strains expressing CreA^HA^ and CreA^GFP^ are phenotypically similar to the wild type on different growth media and retain CCR (Fig. S2).

10.1128/mbio.03734-21.2 FIG S2 Growth phenotype of the wild-type and HA- and GFP-tagged CreA strains grown under different carbon conditions. Plate tests of the wild-type and HA- and GFP-tagged CreA strains grown on solid ANM media containing the indicated carbon compound as the sole carbon source at 37°C for 3 days. Download FIG S2, PDF file, 1.1 MB. Copyright © 2022 Chen et al. 2022 Chen et al. https://creativecommons.org/licenses/by/4.0/ This content is distributed under the terms of the Creative Commons Attribution 4.0 International license.

**FIG 2:**
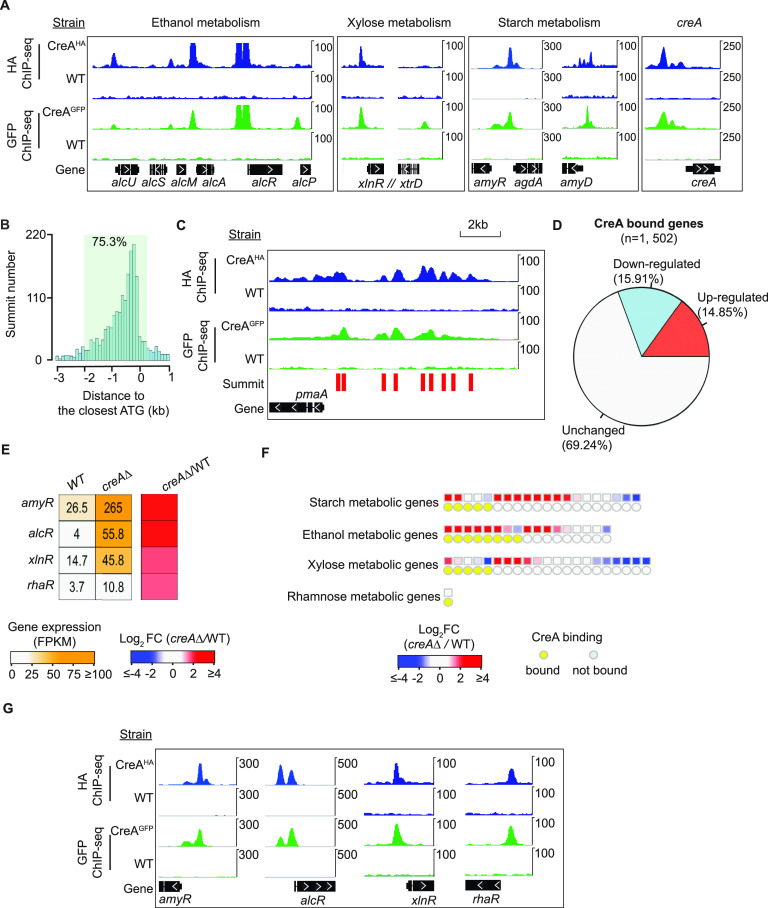
ChIP-seq analysis reveals direct CreA binding to thousands of genomic locations under CCR conditions. (A) Genome browser screenshots showing CreA^HA^ (in blue) and CreA^GFP^ (in green) ChIP-seq signals at the promoter of ethanol, xylose, and starch metabolism genes and the *creA* gene. ChIP signals in wild-type nontagged strains were shown as a negative control. The upper display threshold set on the genome browser for each screenshot is indicated by the scale bar on the right. (B) A bar chart showing the distribution of CreA binding summits at promoters. The distance between each summit to the translation start site (ATG) of the closest gene annotation is presented. (C) A genome browser screenshot showing CreA^HA^ (in blue) and CreA^GFP^ (in green) ChIP-seq signals and peak summits (marked by red bars) identified by MACS2 at the *pmaA* promoter. The upper display threshold set on the genome browser for each screenshot is indicated by the scale bar on the right. (D) A pie chart showing the percentages of CreA-bound genes with different transcriptional consequences. (E) A heatmap plot showing gene expression values and changes (in the *creA*Δ mutant relative to the wild type [WT]) for a few established CreA-bound transcription factors regulating carbon metabolism. (F) Heatmaps displaying gene expression changes for starch, ethanol, xylose, and rhamnose metabolism genes in the *creA*Δ mutant relative to the WT. The presence of CreA binding at their promoters is indicated by a yellow circle. (G) Genome browser screenshots showing CreA^HA^ (in blue) and CreA^GFP^ (in green) ChIP-seq signals at the promoter of *amyR*, *alcR*, *xlnR*, and *rhaR*. The *amyR*, *alcR*, and *xlnR* screenshots are reproduced from panel A, showing only the ChIP-seq signals for these genes of interest. The upper display threshold set on the genome browser for each screenshot is indicated by the scale bar on the right.

Peak-calling analysis by MACS2 (model-based analysis of ChIP-seq version 2) identified 1,988 and 1,717 target sites for CreA^HA^ and CreA^GFP^, respectively (Data Set S3 at https://figshare.com/s/9f324a2370691f209579). On a genome-wide scale, the two different tagged versions of CreA shared highly similar occupancy profiles, with over 70% of target sites in common (Fig. S3A and B). Strong CreA^HA^ and CreA^GFP^ occupancies were observed at the promoter of well-established CreA repressed targets, such as ethanol, xylose, and starch metabolism genes (23, 40, 51, 52) as well as at its own promoter (Fig. 2A). Control analyses suggest that the minor differences in CreA^HA^ and CreA^GFP^ occupancy profiles are due to variations in ChIP efficiency with the different epitopes and/or antibodies (see Materials and Methods). Therefore, the profiles of both CreA^HA^ and CreA^GFP^ were combined to give a total of 2,182 CreA target sites for subsequent analysis (Fig. S3C and Data Set S3).

10.1128/mbio.03734-21.3 FIG S3 ChIP-seq analysis of CreA. (A) A genome browser view of CreA^HA^ (in blue) and CreA^GFP^ (in green) ChIP-seq signals over chromosome III of A. nidulans. ChIP-seq signals in nontagged wild-type strains are presented as negative controls for the corresponding ChIP-seq experiments. Annotated genes are indicated by gray lines at the bottom. (B) A Venn diagram showing the overlap of CreA binding sites detected in CreA^HA^ and CreA^GFP^ strains. Numbers of genes are indicated. (C) Heatmaps displaying CreA^HA^ and CreA^GFP^ ChIP-seq and background (WT) signals over CreA binding sites identified in the CreA^HA^ and CreA^GFP^ strains. The signals across a 3-kb window spanning each CreA ChIP-seq summit are displayed. (D) A diagram showing the frequency of SYGGRG motif across 1-kb regions spanning CreA binding sites. (E) A boxplot displaying CreA ChIP-seq signals at CreA binding sites with or without SYGGRG motif. Background ChIP-seq signal in an untagged wild-type strain grown under glucose conditions was included as a control. (F) Genome browser screenshots showing CreA ChIP-seq signals at representative CreA binding sites with or without the SYGGRG motif. The genomic locations of SYGGRG, motif 2, and motif 4 are indicated on the genome browser by red, blue, and purple markings, respectively. (G) The output of *de novo* motif analysis by MEME-ChIP on CreA binding sites. (H) An illustration showing the presence or absence of SYGGRG and motifs 2 and 4 at each of the CreA binding sites. Download FIG S3, PDF file, 2.9 MB. Copyright © 2022 Chen et al. 2022 Chen et al. https://creativecommons.org/licenses/by/4.0/ This content is distributed under the terms of the Creative Commons Attribution 4.0 International license.

*De novo* motif discovery analysis by MEME-ChIP (Multiple Expectation Maximization for Motif Elicitation for ChIP-seq) identified the previously established CreA DNA recognition motif SYGGRG (10, 13) around peak summit regions (Fig. S3D). Of note, only about half of the CreA target sites (1,104 of 2,182) contain the SYGGRG sequence. CreA target sites with and without the SYGGRG motif have indistinguishable ChIP-seq signals (Fig. S3E) and peak shapes (Fig. S3F), suggesting those detected ChIP-seq peaks without the motif are unlikely to be false positives. MEME-ChIP analysis found three other enriched motifs (Fig. S3G), two of which (motifs 2 and 4) closely resembled the CreA recognition sequence with a core sequence of CGGG and are found at the summit of those target sites without SYGGRG (Fig. S3F). The majority (2,111 out of 2,182) of CreA target sites contain one of the three motifs (Fig. S3H), indicating that the CGGG core is an important element for CreA binding.

About 75% (1,643/2,182) of CreA ChIP-seq peak summits are located at proximal promoter regions (−2,000 to +200 bp with respect to the ATG of corresponding genes) (Fig. 2B), while ∼13% (292/2,182) are in distal upstream intergenic regions (beyond −2,000 bp upstream of ATG) (Fig. S4A). Using only summits located within proximal promoter regions, 1,502 genes were identified as CreA-bound targets under CCR conditions (Data Set S3). Interestingly, almost 40% of CreA target promoters (*n* = 556) showed more than one CreA binding site (Fig. S4B and Data Set S3), with some genes having as many as 8 to 9 target sites (Fig. 2C and Fig. S4C). This suggests that CreA is recruited to multiple locations at a large number of promoters. Taken together, the ChIP-seq results have identified genome-wide CreA occupancies under CCR conditions to a high degree of confidence.

10.1128/mbio.03734-21.4 FIG S4 CreA binds to multiple sites at many promoters. (A) A pie chart showing the percentage of CreA binding sites located at the promoter, gene body, and 3′ end of genes and other genomic regions. (B) Heatmaps displaying CreA ChIP-seq and background (WT) signals over promoters found to have single (top) or multiple (bottom) CreA binding sites. (C) Genome browser screenshots showing ChIP-seq signals at the promoter of representative genes with multiple CreA binding sites in the CreA^HA^, CreA^GFP^, and untagged wild-type control strains. Download FIG S4, PDF file, 1.4 MB. Copyright © 2022 Chen et al. 2022 Chen et al. https://creativecommons.org/licenses/by/4.0/ This content is distributed under the terms of the Creative Commons Attribution 4.0 International license.

### Only a small subset of CreA target genes was derepressed in the *creA*Δ mutant under CCR conditions.

Only 223 out of 1,502 CreA target genes were derepressed in the absence of CreA under CCR conditions, while the expression of the majority of CreA targets (*n* = 1,039) was not significantly altered (less than 2-fold) (Fig. 2D). As mentioned above, the derepression level of some CreA-repressed genes can require not only the relief of CreA-mediated repression but also activation by a pathway-specific transcription factor(s). Consistent with this, genes encoding the transcriptional activators (*amyR*, *alcR*, *xlnR*, and *rhaR*) for the strongly derepressed starch, ethanol, xylan, and rhamnose metabolism genes (23, 40, 51, 53) were significantly upregulated in the *creA*Δ mutant compared to the wild type (Fig. 2E and F). It is noteworthy that those activator genes themselves were also subjected to direct CreA repression, as indicated by the strong CreA occupancy at their promoters (Fig. 2E and G). Therefore, these observations suggest that the lack of derepression for the bulk of the CreA target genes is because the activators for those genes were either not expressed or nonfunctional (likely due to the lack of a small-molecule inducer) under the experimental conditions studied, highlighting the value of the ChIP-seq method in identifying global functions of repressor proteins.

### RNA-seq and ChIP-seq analysis reveals direct and indirect roles for CreA in pathways not directly related to carbon metabolism.

**FIG 3:**
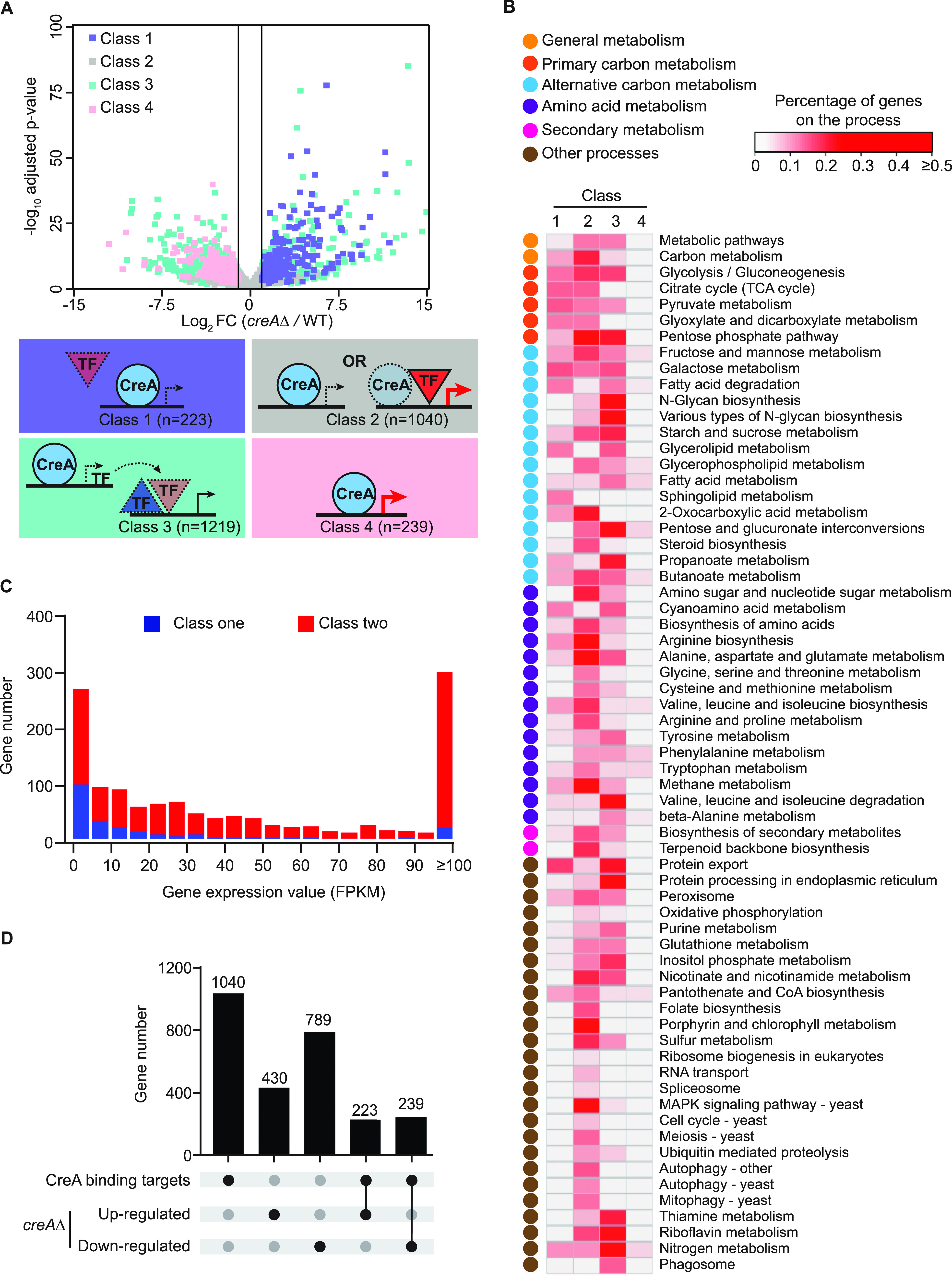
Integrated ChIP-seq and RNA-seq analysis delineates direct and indirect CreA roles on diverse physiological processes beyond carbon metabolism. (A) A schematic diagram illustrating the different regulatory situations for CreA target genes based on the ChIP-seq and RNA-seq results. The genes were grouped into four classes according to the presence or absence of CreA binding at their promoters and the transcriptional effect in the *creA*Δ mutant compared to the wild type (WT), as shown in the volcano plot at the top. Class 1 genes are subjected to CreA direct repression in the wild type and induced by activators (TF, red triangle) in the *creA*Δ mutant. Class 2 genes may contain two groups; one group is repressed by CreA in the wild type, but their expression does not increase in the *creA*Δ mutant due to the absence of transcriptional activator. The other group is activated by activators (red triangle) whose activating function can overwhelm CreA repressive effects, and consequently these genes are expressed at high levels in the wild type and do not have much more induction (e.g., less than 2 folds), if any, in the *creA*Δ mutant. Class 3 genes are not directly controlled by CreA, but their expression levels are up- or downregulated in the *creA*Δ mutant due to derepression of transcriptional activator (red triangle) and repressor (blue triangle) genes, respectively, that are controlled by CreA. Class 4 genes are directly bound by CreA, and their high levels of expression in the wild type require CreA. The different classes of genes and their corresponding transcriptional effects in the *creA*Δ mutant are color-coded similarly in the bottom and top panels, respectively. (B) An overview showing the enriched KEGG pathways in the four classes of genes identified in panel A. The KEGG pathways were broadly classified into six categories, indicated by the different colored dots. The percentage of genes enriched in each KEGG pathway was calculated and is presented in the heatmap. (C) A stacked bar chart displaying the gene expression levels for the genes in class one (blue) and two (red). (D) An UpSet plot showing the overlaps between CreA direct binding and up- and downregulated gene targets.

To identify the overall global functions of CreA and define direct and indirect effects of CreA, an integrated analysis of the RNA-seq and ChIP-seq data was undertaken. Genes were grouped into four classes based on whether their promoters were occupied by CreA (direct targets) and whether their expression was differentially affected by loss of CreA function in the *creA*Δ mutant (direct and indirect targets). The different classes of genes are most likely subjected to different transcriptional regulation, as illustrated in Fig. 3A.

Class 1 genes are directly repressed by CreA in the wild type and induced by activators (e.g., pathway-specific transcription factors) in the *creA*Δ mutant (Fig. 3A). This class of genes is mainly involved in various metabolic pathways, such as glycolysis, gluconeogenesis, pentose phosphate pathway, tricarboxylic acid (TCA) cycle, and metabolism of pyruvate, beta-glucan, starch, galactose, arabinose, and ethanol (Fig. 3B and Data Set S4 at https://figshare.com/s/9f324a2370691f209579). This is consistent with the well-established repressive role of CreA on carbon metabolism. In addition, this class also includes genes with functions in amino acid (*n* = 16) and secondary (*n* = 20) metabolism.

Genes in class 2 also showed CreA binding at their promoters, but their gene expression was not dramatically increased (e.g., less than 2-fold) in the absence of CreA function. This may be due to two possible regulatory situations (Fig. 3A). In the first one, the transcriptional activators of the CreA-bound genes were absent under the studied experimental conditions (1% glucose), such that depression could not occur even in the *creA*Δ mutant. The other possible situation is that the transcriptional activators of these CreA-bound genes were highly active and thereby overwhelming the CreA repressive effect. Consequently, these genes were expressed at high levels in both the wild type and the *creA*Δ mutant with no significant change in gene expression. Consistent with these possibilities, both highly and lowly expressed genes (i.e., with and without transcriptional activation, respectively) were found for class 2 genes in the wild type, in contrast to the majority of class 1 genes being lowly expressed (Fig. 3C). Regardless of the exact activation situation, the occupancy of CreA at these promoters indicates a direct role of CreA for this class of genes, which are enriched with almost the same Kyoto Encyclopedia of Genes and Genomes (KEGG) pathways (e.g., central carbon, alternative carbon, amino acid, and secondary metabolism pathways) as those described for class 1. Notably, for most of the common metabolic pathways, there were actually more genes belonging to class 2 than to class 1 (Fig. 3B and Data Set S5 at https://figshare.com/s/9f324a2370691f209579). In addition, several KEGG pathways are largely unique to the class 2 genes, such as “MAPK signaling pathway” (*n* = 16), “Meiosis” (*n* = 8), “Autophagy” (*n* = 7), “RNA transport” (*n* = 6), “Cell cycle” (*n* = 5), and “Spliceosome” (*n* = 4), uncovering unknown potential direct roles of CreA in these cellular processes.

For class 3 genes, no CreA occupancy was detected at their promoters, but expression of these genes was differentially altered by the loss of CreA function. The transcriptional changes are, therefore, caused by an indirect role of CreA. The indirect effect may be the result of derepression of transcriptional activator or repressor genes that are controlled by CreA (Fig. 3A), altered physiologies due to deregulated metabolic flux, and/or the extreme growth defect of the *creA*Δ mutant. It is noteworthy that this class of genes represents about 75% of the transcriptional effects observed in the *creA*Δ mutant (Fig. 3D), indicating a great deal of CreA functions are indirect. Similar to the above-described two classes, many genes from class 3 have functions in various carbon metabolic pathways as well as in amino acid and secondary metabolism, indicating extensive direct and indirect control by CreA in both primary and secondary metabolism (Fig. 3B). In addition, genes for the KEGG pathways of “Protein processing in endoplasmic reticulum,” “N-Glycan biosynthesis,” “Phagosome,” and “Valine, leucine and isoleucine degradation” were affected mostly by indirect CreA regulation (e.g., 26 out of 34, 13 out of 15, 5 out of 6, and 12 out of 17 CreA-controlled genes, respectively) (Data Set S6 at https://figshare.com/s/9f324a2370691f209579).

The last class of CreA-bound genes (*n* = 239; class 4) was positively regulated by CreA (e.g., downregulated in the *creA*Δ mutant) (Fig. 3A). This class of genes is controlled by either indirect CreA regulation or a previously unknown activator function of CreA. At the moment, we cannot distinguish between the two possibilities. Other than a few genes belonging to the KEGG “Metabolic pathways” (*n* = 16) and “Biosynthesis of secondary metabolites” (*n* = 5) categories, this class of genes is not significantly enriched for any pathway or process (Fig. 3B and Data Set S7 at https://figshare.com/s/9f324a2370691f209579).

Taken together, the integrated analysis of RNA-seq and ChIP-seq has uncovered the direct and indirect roles of CreA over diverse processes, providing insights into the regulation of CreA targets.

### Evidence for CreA functions in secondary metabolism, iron homeostasis, and oxidative stress response.

**FIG 4:**
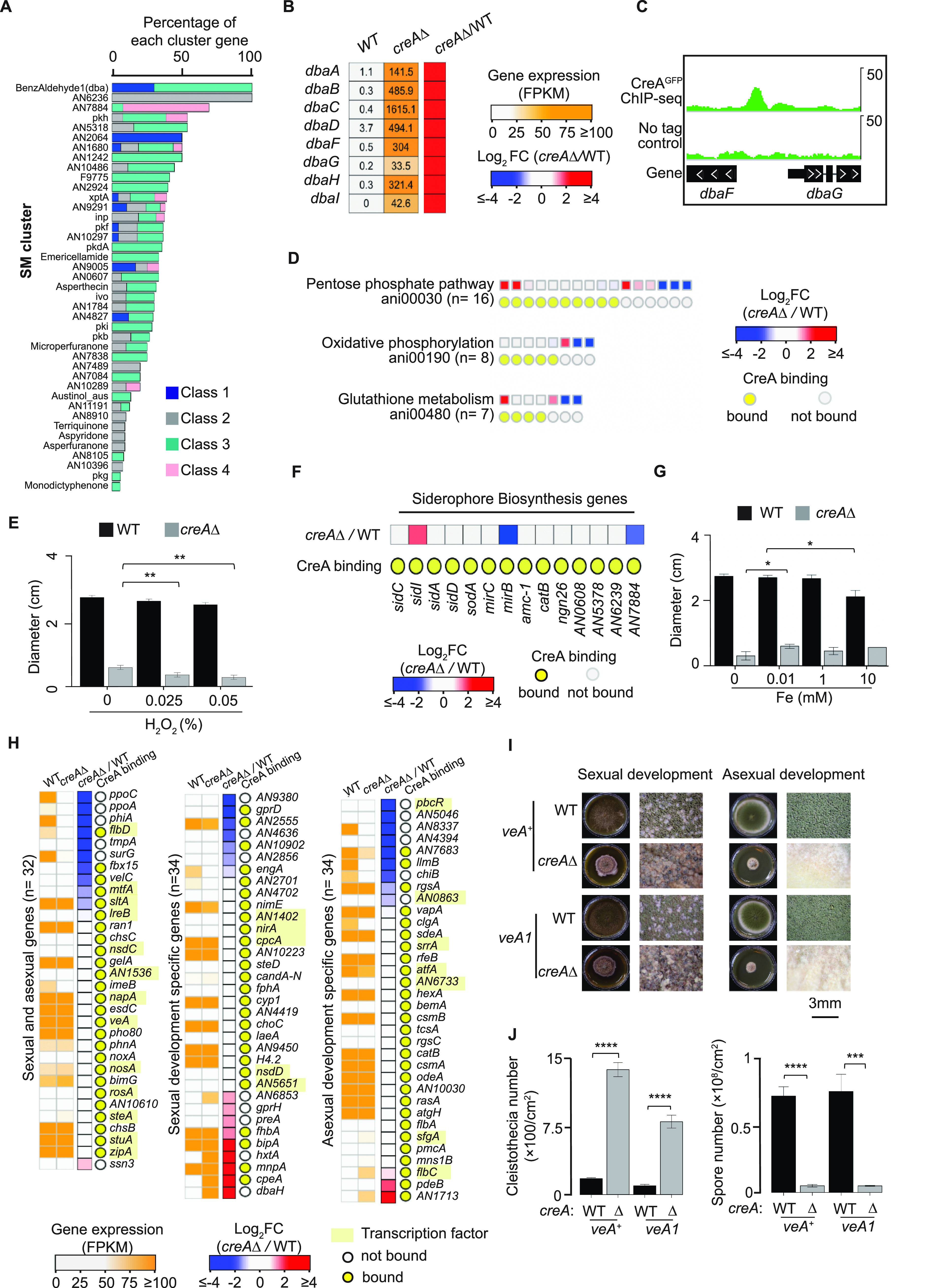
CreA regulates genes involved in secondary metabolism, iron homeostasis, oxidative stress, and asexual and sexual developmental processes. (A) A bar plot showing percentage of genes from the four regulatory situations (class 1 to 4) identified from the integrated analysis for each secondary metabolism (SM) cluster. (B) A heatmap plot showing gene expression values and changes (in the *creA*Δ mutant relative to wild type [WT]) for the BenzAldehyde1 (dba) cluster genes. (C) A genome browser screenshot showing CreA ChIP-seq signals at the divergent promoters of *dbaF* and *dbaG*. The upper display threshold set on the genome browser for each screenshot is indicated by the scale bar on the right. (D and F) Heatmap plots displaying gene expression changes for pentose phosphate pathway, oxidative phosphorylation, and glutathione (D) and siderophore biosynthesis genes (F) in the *creA*Δ mutant relative to WT. The presence of CreA binding at their promoters is indicated by a yellow circle. (E and G) Bar plots showing the growth diameter of the wild type and the *creA*Δ mutants under conditions with the indicated hydrogen peroxide (H_2_O_2_) (E) and iron (Fe) concentrations (G). *t* test was used to determine statistical significance. *, *P* ≤ 0.05; **, *P* ≤ 0.01. (H) Heatmap plots showing gene expression values and changes (in the *creA*Δ mutant relative to the WT) for sexual and asexual developmental genes. The presence of CreA binding at the promoters is indicated by a yellow circle, and transcription factor genes are highlighted in yellow. (I) Photos showing the sexual and asexual phenotypes of the wild type and the *creA*Δ mutant. (J) Bar graphs present the quantification of cleistothecia and spores of the wild type and the *creA*Δ mutant shown in panel I. *t* test was used for the *P* values. ***, *P* ≤ 0.001; ****, *P* ≤ 0.0001.

Notably, 131 genes from the integrated analysis are annotated with the KEGG pathway “Biosynthesis of secondary metabolite,” with 40 and 91 being indirect and direct targets, respectively. Among the direct targets, over three-quarters of them (*n* = 66) did not change their expression in the *creA*Δ mutant (Data Set S8 at https://figshare.com/s/9f324a2370691f209579), suggesting that their specific transcriptional regulator was either absent or not functional. This is consistent with the silent nature of secondary metabolism genes during the primary metabolism growth stage. Mapping of both direct and indirect target genes to previously annotated secondary metabolism clusters showed that the majority of the clusters (41 out of 51) had at least one gene bound by CreA (Fig. 4A). Of note, 8 of the 10 biosynthesis genes (e.g., *dbaA*, *dbaB*, *dbaC*, *dbaD*, *dbaF*, *dbaG*, *dbaH*, and *dbaI*) for the secondary metabolite 2,4-dihydroxy-3-methyl-6-(2-oxopropyl) benzaldehyde (dba) were significantly upregulated in the *creA*Δ mutant compared to the wild type (Fig. 4B). A strong CreA binding peak was found at the divergent promoter of *dbaF* and *dbaG* (encoding the *dba* cluster-specific transcription factor) but not at other *dba* genes (Fig. 4C).

The functional enrichment analysis also identified pentose phosphate pathway (*n* = 16), oxidative phosphorylation (*n* = 8), and glutathione metabolism (*n* = 7) as being under CreA control. While about half of these genes had their expression altered in the *creA*Δ mutant (*n* = 15), the others (*n* = 16) were only bound by CreA without any transcriptional effect (Fig. 4D), possibly because the cells were not experiencing any oxidative stress under the studied growth conditions. Growth analysis showed that the *creA*Δ mutant is more sensitive to H_2_O_2_ than the wild type (Fig. 4E). Although the growth defect of the mutant may have compromised the sensitivity of this growth assay (Fig. S5A), the growth reduction was statistically significant and supports the finding from the integrated ChIP-seq and RNA-seq analysis that CreA targets genes important for oxidative stress response.

10.1128/mbio.03734-21.5 FIG S5 Phenotypic characterization of the *creA*Δ mutant. (A and D) Representative photos for the plate tests presented in Fig. 4E and G for the phenotypes of the wild type and the *creAΔ* mutant grown on solid media containing different (A) hydrogen peroxide (H_2_O_2_) and (D) iron (Fe) concentrations at 37°C for 3 days, respectively. (B) A genome browser screenshot for CreA ChIP-seq signal at the *sreA* gene promoter. ChIP signals in a wild-type nontagged strain is shown as a negative control. The upper display threshold set on the genome browser for each screenshot is indicated by the scale bar on the right. (C) A boxplot showing the gene expression values of *sreA* in wild type and the *creAΔ* mutant. (E) Photos showing the sexual and asexual phenotypes of strains expressing CreA from the native (WT) or overexpressing *gpdA* [*gpdA*(p)*creA*] promoters. The bar graphs at the bottom present the quantification of cleistothecia and spores observed for each strain after growing at 37°C for 10 days in the dark and 3 days in the presence of light, respectively. *t* test was used for the *P* values. **, *P* ≤ 0.01. Download FIG S5, PDF file, 1.2 MB. Copyright © 2022 Chen et al. 2022 Chen et al. https://creativecommons.org/licenses/by/4.0/ This content is distributed under the terms of the Creative Commons Attribution 4.0 International license.

In addition, several genes associated with siderophore biosynthesis and transport (e.g., *sidC*, *sidI*, *sidA*, *sidD*, *sodA*, *mirC*, *mirB*, *amc-1*, *catB*, *ngn26*, *AN0608*, *AN5378*, *AN6239*, and *AN7884*) were also bound by CreA but without any expression change in the *creA*Δ mutant (Fig. 4F). It is interesting that the *sreA* gene, which encodes the major negative regulator of iron homeostasis (54), is also a direct target of CreA (Fig. S5B and C). Growth analysis showed that the growth of the wild type is slightly sensitive to high concentrations of iron (e.g., 10 mM), while the *creA*Δ mutant did not seem to be affected despite its severe growth defect (Fig. 4G and Fig. S5D). On the other hand, the *creA*Δ mutant was actually less capable of growing in the absence of iron compared to its growth on media supplemented with iron. This is in contrast to the wild-type strain that had comparable growth under both conditions (Fig. 4G and Fig. S5D), supporting a role for CreA in iron acquisition and homeostasis.

### CreA controls asexual and sexual developmental genes and processes.

The integrated analysis also identified a larger number of genes (*n* = 100; Data Set S9 at https://figshare.com/s/9f324a2370691f209579) belonging to the asexual and sexual developmental processes, including the developmental regulatory genes *steA*, *stuA*, *nsdC*, and *flbD*. Notably, about half of these genes were not differentially expressed in the *creA*Δ mutant compared to the wild type (Fig. 4H), most likely because the experiment was performed under conditions (submerged liquid culture) that suppress development; hence, the developmental regulators were not functional. To confirm that CreA indeed affects development, the asexual and sexual development phenotypes of the wild type and the *creA*Δ mutant were analyzed. Light is the key environmental signal in determining asexual and sexual development in filamentous fungi (55), and the light-responsive regulation is mediated by VeA in A. nidulans (56). The strains used in both ChIP-seq and RNA-seq experiments carry a *veA1* allele that results in a light-insensitive phenotype. To exclude any potential effect of *veA1* on CreA, we generated *creA*Δ mutants in both *veA1* and *veA^+^* backgrounds by genetic crossing. In the presence of light, the number of conidia (asexual development) was significantly reduced in the absence of *creA* for both *veA* backgrounds (Fig. 4I and J). This is not due to a relatively lower growth rate of the mutant, as conidiation is still largely impaired even after extended growth (10 days). On the other hand, the *creA*Δ mutants in both *veA1* and *veA^+^* backgrounds produced significantly increased numbers of sexual fruiting bodies (cleistothecia) compared with the corresponding wild-type strains (Fig. 4I and J). Consistent with this, there was increased and decreased asexual and sexual development, respectively, in a strain overexpressing CreA from the *gpdA* promoter compared to the wild type (Fig. S5E). Taken together, these results indicate a role for CreA in promoting conidiation (i.e., asexual development) and suppressing sexual development through directly and indirectly controlling both upstream regulators and downstream developmental genes.

### CreA regulates diverse transporter functions.

**FIG 5:**
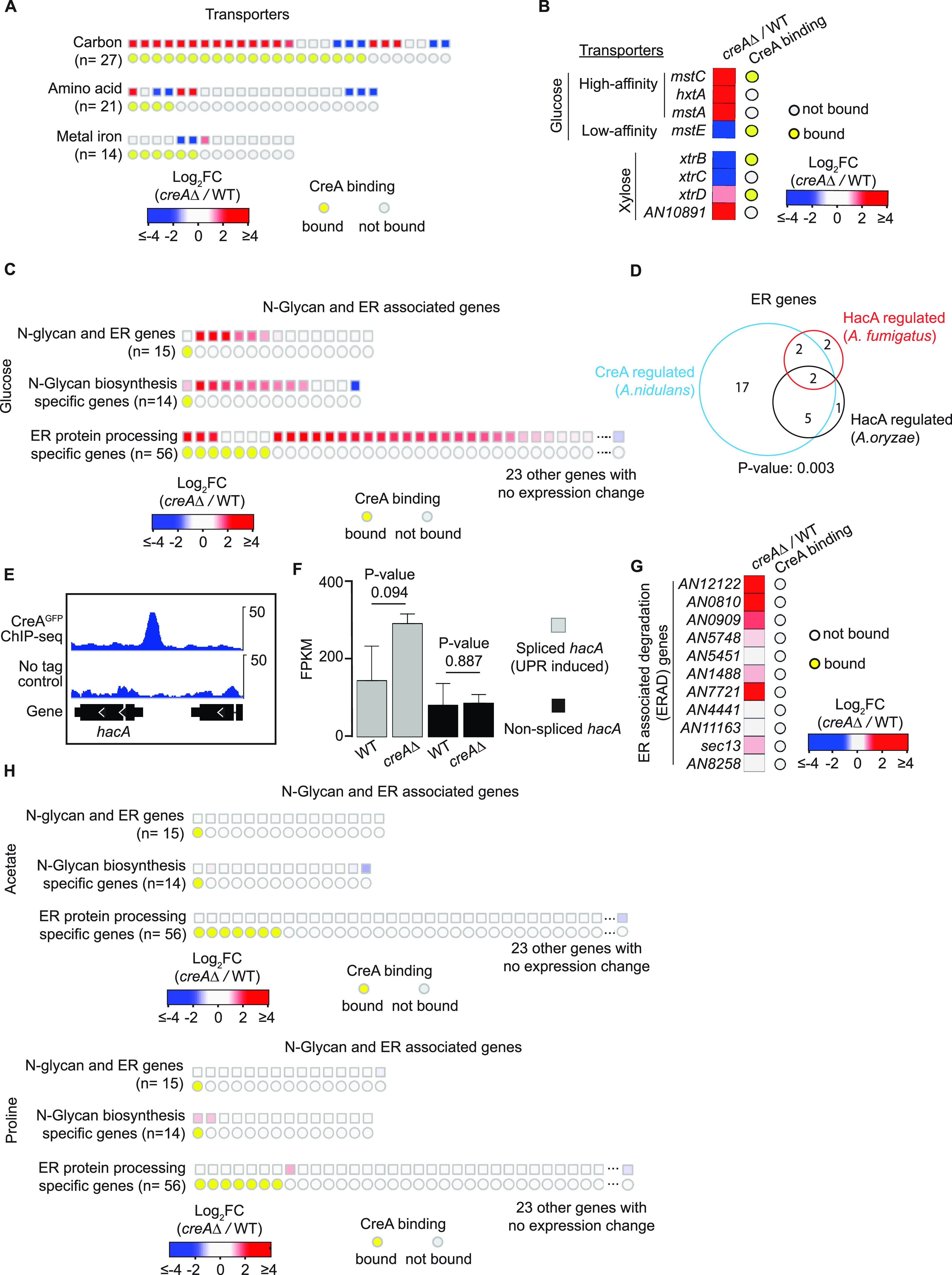
CreA regulates genes involved in the unfolded protein response, nutrient transport, and filamentous fungal specific functions. (A, B, C, and G) Heatmap plots displaying gene expression changes for transporter genes (A and B), N-glycan and ER-associated genes (C), and ER-associated degradation (ERAD) genes (G) in the *creA*Δ mutant relative to the wild type (WT) under glucose culture conditions. The presence of CreA binding at their promoters is indicated by a yellow circle. (D) A Venn diagram showing the overlap of CreA-regulated ER-associated genes in A. nidulans and HacA-regulated genes in A. fumigatus and A. oryzae. (E) A genome browser screenshot for CreA ChIP-seq signals at the *hacA* promoter. The upper display threshold set on the genome browser for each screenshot is indicated by the scale bar on the right. (F) A bar plot showing gene expression values (FPKM) of spliced *hacA* and nonspliced *hacA* in the WT and the *creAΔ* mutant. The level of spliced *hacA*, which is activated by unfolded protein stress, is presented in gray, while the level of nonspliced *hacA* is shown in black. Error bars display the FPKM standard deviation between biological repeats. (H) Heatmap plots displaying gene expression changes for N-glycan and ER-associated genes when acetate or proline was used as the sole carbon source.

The GO biological process “transmembrane transport” is also significantly enriched (*P*  = 3.17 × 10^−9^; Data Set S10 at https://figshare.com/s/9f324a2370691f209579) among CreA-occupied targets and differentially expressed genes (DEGs). A total of 288 transporter-encoding genes were bound by CreA and/or differentially expressed in the *creA*Δ mutant (Data Set S10). These genes are involved in the transport of carbohydrates (*n* = 24), amino acids (*n* = 9), ions (e.g., cation, anion, and metal ions) (*n* = 7), and drugs (*n* = 10) (Data Set S10). It is noteworthy that the various transporter genes were differently affected by CreA (Fig. 5A). For example, CreA mainly acted directly and negatively on carbohydrate transporter genes (e.g., *mstC*, *hxtA*, *mstA*, *xtrD*, *AN9173*, *lacpA*, *AN4590*, *AN1797*, *AN2814*, *AN3515*, and *AN8347*), while the expression of the various amino acid and metal ion transporter genes was affected differently by wild-type CreA functions (Fig. 5A). Moreover, we also found evidence for CreA determining the type of transporter for a given substrate to express in the cell, e.g., CreA repressed the expression of the high-affinity glucose transporters (e.g., *mstC*, *hxtA*, and *mstA*) in the wild type while positively affecting the low-affinity glucose transporter gene *mstE* (Fig. 5B). Similar opposite effects of CreA on the expression of xylose transporter genes were also observed, e.g., *xtrD* and *AN10891* were negatively controlled by CreA, while *xtrB* and *xtrC* expression was dependent on wild-type CreA functions (Fig. 5B). Overall, these observations suggest a critical role for CreA in controlling the influx and efflux of sugar substrates, amino acids, ions, and drugs.

### The *creA*Δ mutant experiences unfolded protein stress when growing on glucose.

Class 3 genes (e.g., indirectly controlled by CreA) are enriched with two KEGG pathways (“Protein processing in ER” and “N-Glycan biosynthesis”) related to endoplasmic reticulum (ER) functions. The two sets of genes are partially overlapping, and most of their expression was induced in the *creA*Δ mutant (Fig. 5C), suggesting the activity of a transcriptional activator for the ER functions can be activated in the absence of CreA function. In A. fumigatus and A. oryzae, expression of ER-associated genes is modulated by HacA, which is a highly conserved transcriptional activator of unfolded protein response genes (57–61).

Those ER-associated genes upregulated in the *creA*Δ mutant largely overlap the A. fumigatus and A. oryzae HacA-dependent ER-associated genes (Fig. 5D). Moreover, the A. nidulans *hacA* gene is a direct binding target of CreA and was upregulated in the *creA*Δ mutant (Fig. 5E and F). Therefore, HacA is likely responsible for the induction of those ER-associated genes in the *creA*Δ mutant. However, transcriptional upregulation of the *hacA* level alone is not sufficient to drive induction of its downstream targets, as HacA transcriptional activity requires induction by a signal from unfolded protein stress through a conserved mechanism involving splicing of a cryptic intron in the *hacA* transcript (62). Inspection of the splicing of the cryptic intron on the *hacA* transcript showed a 2-fold increase in the spliced form of *hacA* in the *creA*Δ mutant compared to the wild type, while the levels of unspliced *hacA* transcript did not change (Fig. 5F). Moreover, several ER-associated degradation (ERAD) genes, the expression of which is induced by unfolded protein stress, were also upregulated in the *creA*Δ mutant compared to the wild-type strain (Fig. 5G). These observations suggest that the *creA*Δ mutant experiences unfolded protein stress when growing on glucose. Interestingly, when acetate or proline was used as the sole carbon source, expression of ER-associated and unfolded protein genes was not affected by the loss of *creA* (Fig. 5H). The condition-specific effect implies that the unfolded protein stress experienced by the *creA*Δ mutant is an indirect physiological consequence of the mutant’s phenotype rather than a direct CreA function, consistent with the differential expression of class 3 genes being indirect effects.

### CreA targets many filamentous fungus-specific genes.

**FIG 6:**
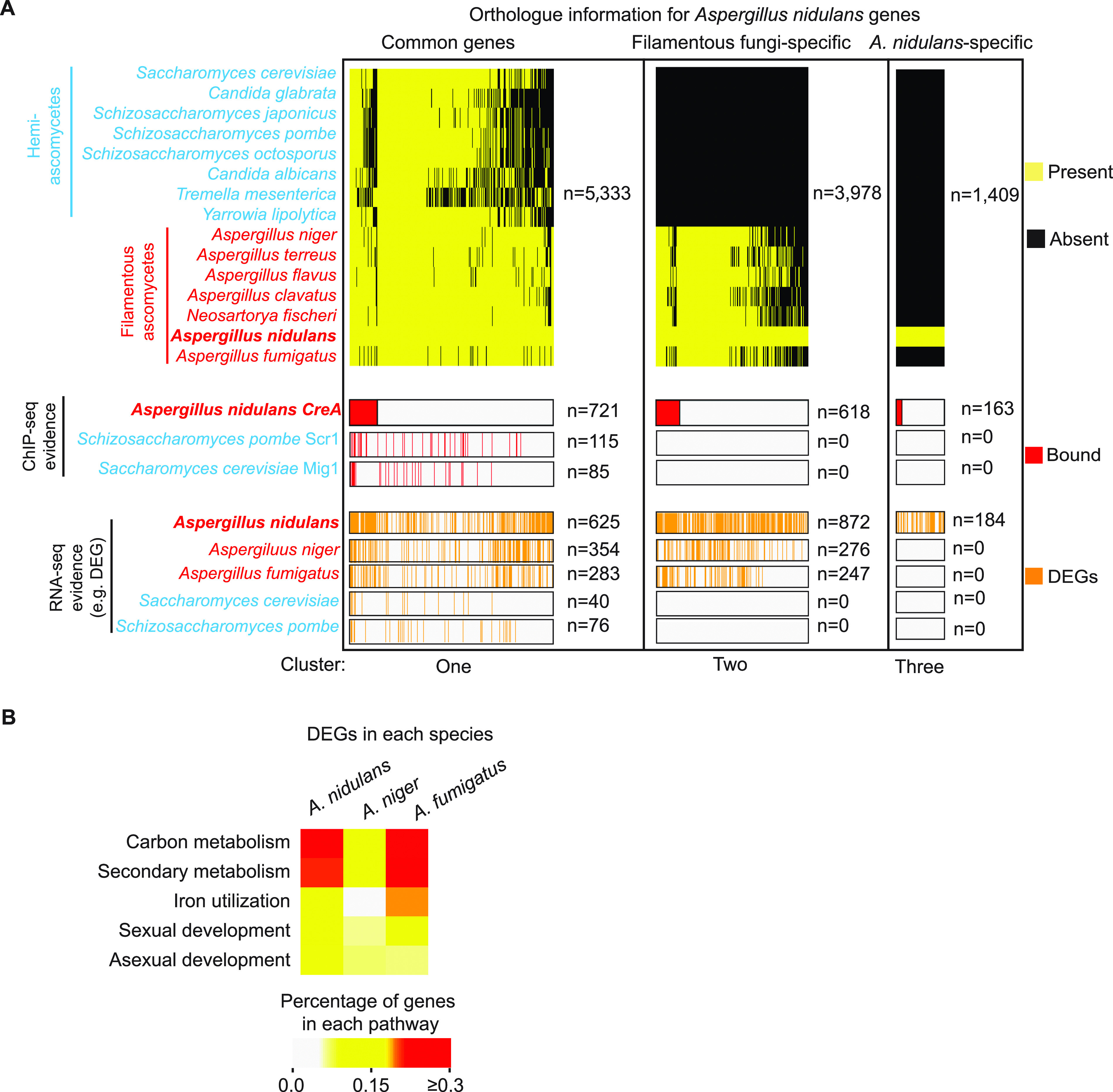
CreA targets many filamentous fungus-specific genes. (A) A summary diagram displaying orthologue information for A. nidulans genes in the indicated hemi-ascomycete and filamentous ascomycete species. The upper panel shows the presence and absence of orthologue for each A. nidulans gene in the indicated species. The A. nidulans genes were separated into three groups, namely, genes shared by most species (common), genes unique to filamentous ascomycetes (filamentous fungus specific), and genes unique to A. nidulans (A. nidulans specific). The middle panel marks the genes bound by CreA, S. pombe Scr1, or S. cerevisiae Mig1 based on ChIP evidence, while the lower panel indicates differentially expressed genes (as determined by RNA-seq) in the mutant of the *creA* homologue of the corresponding species. Gene number for each category is indicated by *n*. (B) Heatmap plot showing the percentage of DEGs enriched in the indicated pathways for the *creAΔ* mutant of the three different species.

It is noteworthy that the number of differentially regulated genes in the S. cerevisiae (6), S. pombe (63), and C. albicans (64) mutants lacking the corresponding CreA orthologue was significantly lower (*n* = 48, 81, and 230, respectively) than the number we observed for the *creA*Δ mutant of A. nidulans (*n* = 1,681) (Fig. S6A). The number of directly bound targets of S. cerevisiae Mig1 (*n* = 113) (65) and S. pombe Scr1 (*n* = 140) (63) was also much lower than that of CreA (*n* = 1,502) (Fig. 6A). Interestingly, phylogenetic analysis of CreA direct target genes revealed more than half (52%) of them are unique to filamentous fungi, with about 10% being unique to A. nidulans (Fig. 6A). Consistent with this, analysis of published transcriptome profiling data (47, 66) (SRA accession number PRJNA500662) found that the CreA homologue of other filamentous fungi (e.g., A. fumigatus, A. niger, and *P. oxalicum*) also have more regulatory targets (*n* = 852, 1,037, and 1,659, respectively) than the yeast homologues (Fig. S6A). Many of those A. fumigatus and A. niger CreA targets are also filamentous fungus-specific genes (*n* = 276 and 247, respectively) (Fig. 6A) and enriched in functional pathways similar to those for CreA (Fig. 6B). Therefore, CreA (and probably its functional homologues in other filamentous fungi) may have expanded its regulatory controls over many filamentous fungus-specific genes whose functions are probably needed for filamentous fungal processes, or perhaps more likely, yeast orthologues may have lost many of CreA functions.

10.1128/mbio.03734-21.6 FIG S6 Number of genes controlled by CreA and its homologues. (A) A bar plot showing the number of up- and downregulated genes in the *creAΔ* mutants compared to the wild type in the indicated fungal species. (B) A bar plot showing the number of CreA binding sites under different carbon conditions. Gene numbers are indicated at the top of the bars. Download FIG S6, PDF file, 0.6 MB. Copyright © 2022 Chen et al. 2022 Chen et al. https://creativecommons.org/licenses/by/4.0/ This content is distributed under the terms of the Creative Commons Attribution 4.0 International license.

### CreA binding to DNA is not determined by CCR signals but depends on its protein level.

**FIG 7:**
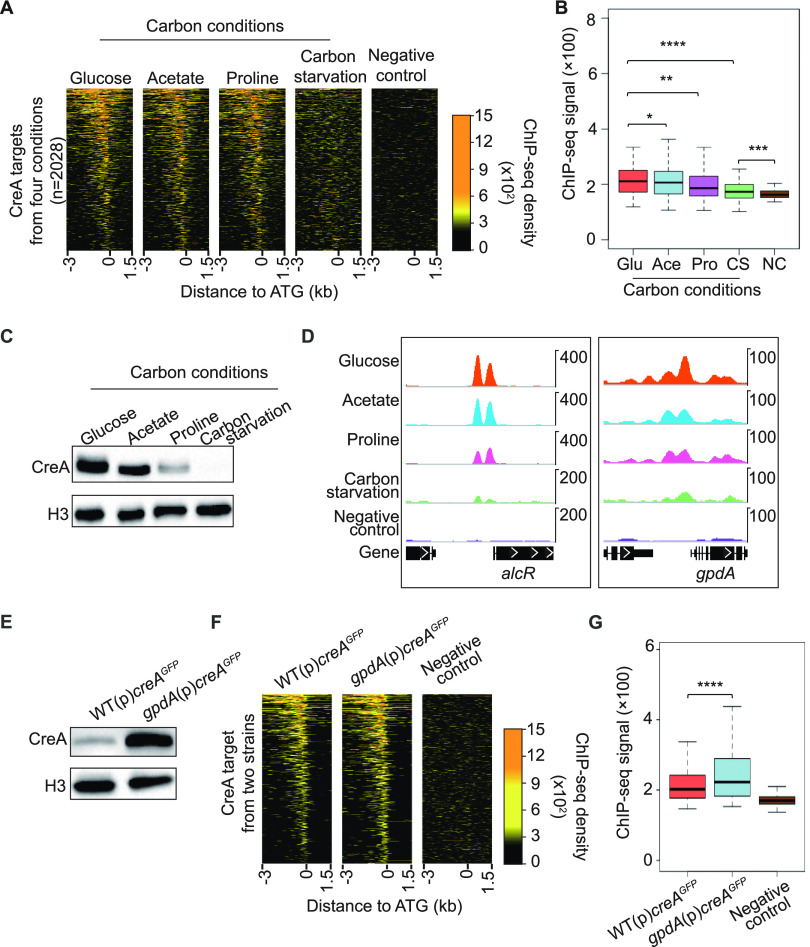
Level of CreA binding to DNA under different carbon conditions is dependent on its protein levels. (A) Heatmap plots showing CreA^HA^ ChIP-seq signals at the promoters of its targets under glucose, acetate, proline, and carbon-free conditions. The background ChIP-seq signal in an untagged wild-type strain grown under glucose conditions was included as a negative control. (B) A boxplot displaying overall CreA ChIP-seq signals for glucose (Glu), acetate (Ace), proline (Pro), and carbon starvation (CS) conditions. Background ChIP-seq signal in an untagged wild-type strain grown under glucose conditions was included as a negative control (NC). *t* test was used for statistical significance. *, *P* ≤ 0.05; **, *P* ≤ 0.01; ***, *P* ≤ 0.001; ****, *P* ≤ 0.0001. (C) Western blot analysis showing CreA expression in the wild type grown under glucose, acetate, proline, and carbon starvation conditions. The level of histone H3 was used as an internal loading control. (D) Genome browser screenshots for CreA ChIP-seq signals at the promoter region of *alcR* and *gpdA* under glucose, acetate, proline, and carbon starvation conditions. The upper display threshold set on the genome browser for each screenshot is indicated by the scale bar on the right. (E) Western blot analysis showing CreA levels in the wild type [WT(p)*creA^GFP^*] and overexpression [*gpdA*(p)*creA^GFP^*] strains grown under glucose conditions. The level of histone H3 was used as an internal loading control. (F) Heatmap plots displaying CreA ChIP-seq signals at the promoter of CreA targets in strains expressing CreA^GFP^ from the native *creA* [WT(p)*creA^GFP^*] or overexpressing *gpdA* [*gpdA*(p)*creA*^GFP^] promoters under glucose conditions. The background ChIP-seq signal in an untagged wild-type strain grown under glucose conditions was presented as a negative control. (G) A boxplot displaying the overall CreA ChIP-seq signals at the promoter of CreA targets in strains expressing CreA^GFP^ from the native *creA* [WT(p)*creA^GFP^*] or overexpressing *gpdA* [*gpdA*(p)*creA^GFP^*] promoters under glucose conditions. *t* test was used for statistical significance. ****, *P* ≤ 0.0001.

The above-described results suggest a much wider role for CreA in diverse physiological processes beyond carbon metabolism. This, together with the growth impairment of the *creA*Δ mutant under both carbon catabolite-repressing and derepressing conditions (Fig. S1A), indicates CreA has critical functions even under partial carbon catabolite derepression conditions. To assess this and determine the scope of CreA functions under other conditions, we performed ChIP-seq against CreA^HA^ under three different growth conditions (e.g., acetate or proline as the sole carbon source or carbon starvation). CreA^HA^ occupancy profiles were highly similar when glucose, acetate, or proline was used as the sole carbon source, only differing in the overall occupancy intensities (Fig. 7A). The occupancy levels correlated with CreA protein levels between these carbon conditions (Fig. 7B and C). Of note, CreA occupancy of many targets (albeit at lower levels) was still evident in carbon-starved cells (Fig. 7A and D) in which CreA protein could not be detected by Western blot analysis (Fig. 7C). This indicates that a small amount of CreA exists in the nucleus binding to target promoters even during carbon starvation conditions. Nevertheless, the number of genome-wide CreA binding sites is much reduced compared to that of the other carbon conditions (Fig. S6B and Data Set S11 at https://figshare.com/s/9f324a2370691f209579).

The similar ChIP-seq profiles under different carbon conditions indicate that CreA occupancy of target sites is mainly dependent on the CreA protein level. To gain additional support, we carried out ChIP-seq against CreA in a strain overexpressing CreA^GFP^ from the constitutively expressed strong promoter of *gpdA* (Fig. 7E). While the overall CreA occupancy profile in the overexpression strain was essentially identical to that of the strain with wild-type CreA expression (Fig. 7F), CreA occupancies were generally higher in the overexpression strain (Fig. 7G). These results demonstrate that CreA protein levels under different CCR conditions affect CreA binding but not target selection in the genome.

### DNA binding competition with pathway-specific factors is not a universal mechanism for CreA repression.

**FIG 8:**
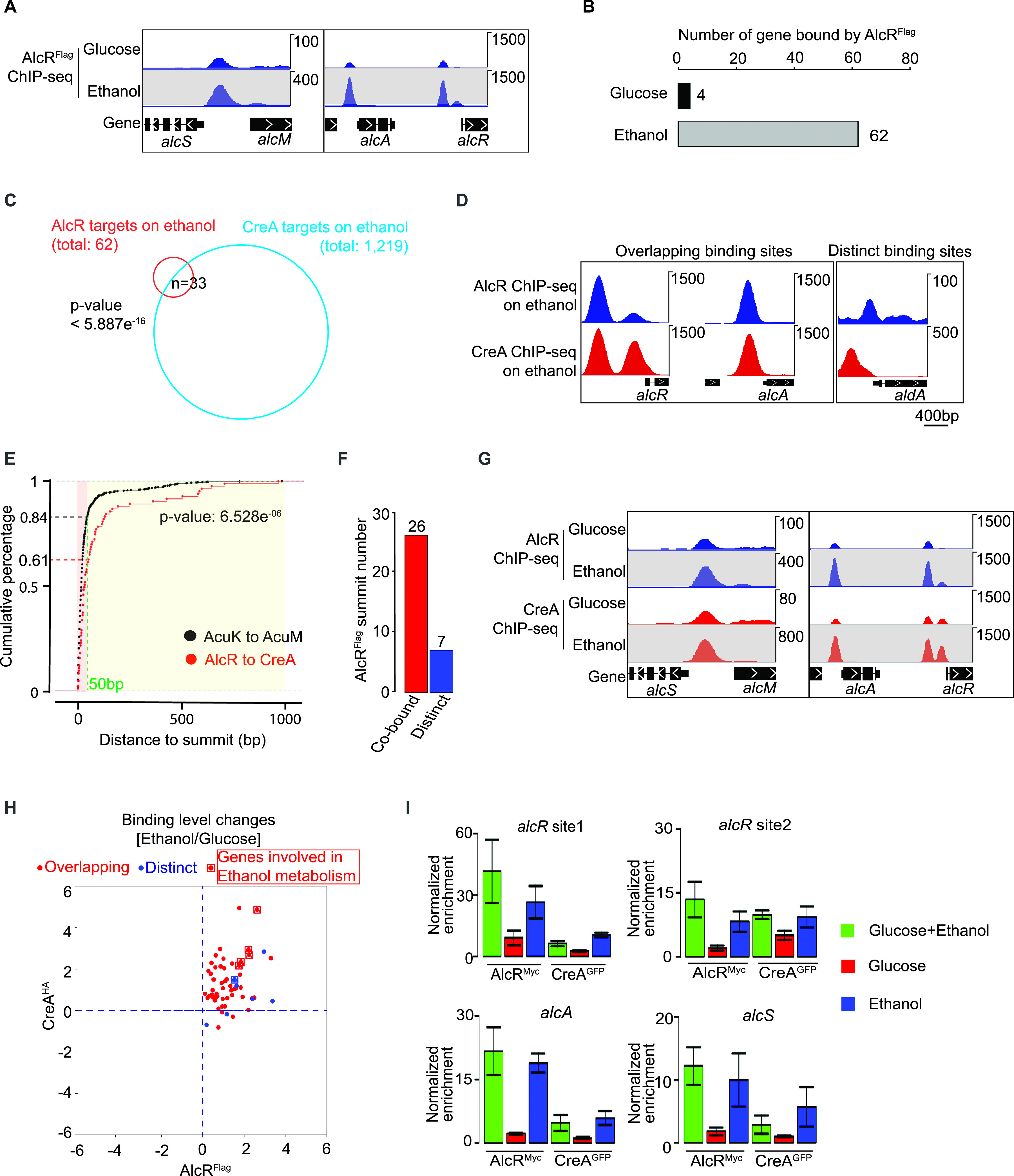
DNA binding competition with pathway specific factors is not a universal mechanism for CreA repression. (A) Genome browser screenshots showing AlcR ChIP-seq signals at the promoter of ethanol metabolism genes under glucose and ethanol conditions. (B) A bar plot showing the number of AlcR binding sites identified by MACS2 under glucose and ethanol conditions. (C) A Venn diagram showing the overlap of CreA and AlcR target genes on ethanol condition. (D) Genome browser screenshots showing AlcR and CreA ChIP-seq signals at select promoter of ethanol metabolism genes on ethanol conditions. The upper display threshold set on the genome browser for each screenshot is indicated by the scale bar on the right. (E) A cumulative plot showing the distributions of distances between AlcR and CreA (red line) and between AcuK and AcuM (black line) ChIP-seq peak summits. The percentages of AlcR-CreA and AcuK-AcuM bound peaks with a distance of less than 50 bp are indicated on the *y* axis. (F) A bar plot showing the number of promoter binding events with AlcR and CreA summits located within 50 bp of each other (Cobound) or greater than 50 bp apart (Distinct). (G) Genome browser screenshots showing CreA and AlcR ChIP-seq binding signals at the promoter of ethanol metabolism genes under glucose and ethanol conditions. The upper display threshold set on the genome browser for each screenshot is indicated by the scale bar on the right. (H) A scatterplot showing binding level changes of CreA and AlcR between glucose and ethanol conditions at their common target promoters. (I) Bar plots showing the result of ChIP-qPCR against CreA and AlcR at the promoter of ethanol metabolism genes in the same CreA^GFP^ AlcR^Myc^ double-tagged strain grown under repressing (glucose), inducing (ethanol), and induced-repressed (glucose and ethanol) carbon conditions.

DNA binding competition with pathway-specific transcription factors is one mechanism for CreA repression, first established for CreA and the pathway-specific factor AlcR using *in vitro* gel mobility shift assay (19). We determined whether this is a general mechanism by comparing genome-wide AlcR and CreA occupancies during growth on repressing (e.g., glucose) and inducing (e.g., ethanol) carbon sources. On glucose, there were weak AlcR^FLAG^ occupancies only at four ethanol metabolism genes (*alcR*, *alcA*, *alcM*, and *alcS*) (Fig. 8A), consistent with previous findings that *alcR* is repressed by CreA and expressed at a basal level during growth on glucose. In contrast, strong distinct AlcR^FLAG^ ChIP-seq peaks were observed at the promoter of these (Fig. 8A) as well as 58 additional genes under ethanol conditions (Fig. 8B and Data Set S12 at https://figshare.com/s/9f324a2370691f209579).

A significant overlap was observed between AlcR and CreA targets (Fig. 8C). Inspection of the ChIP-seq data on a genome browser revealed two different cooccupancy patterns, namely, overlapping and distinct target site occupancies (Fig. 8D). To systematically classify the two cooccupancy patterns, we first performed a control ChIP-seq experiment to measure the distance on DNA between the two subunits of the obligate AcuK-AcuM heterodimer in a double-tagged (AcuK^MYC^ and AcuM^FLAG^) strain (67). As expected for the subunits of an obligate heterodimer binding to the same genomic sites, the AcuK^MYC^ and AcuM^FLAG^ ChIP-seq data are highly correlated (*R* = 0.95) and are indistinguishable from their biological repeats (*R* = 0.94 for AcuK^MYC^ and *R* = 0.96 for AcuM^FLAG^). The ChIP-seq peak summits of AcuK^MYC^ and AcuM^HA^ are adjacent to each other (50 bp or less) for the large majority of their cooccupied sites (Fig. 8E). Using this distance as a guide, we estimated that CreA and AlcR occupy the same sites or are located at very close proximity to each other (e.g., within 50 bp) at ∼80% of cooccupied promoters, while about 20% had CreA and AlcR binding to distinct nonoverlapping sites (e.g., more than 50 bp apart) (Fig. 8F).

Contrary to the expectation for the DNA binding competition model, *in vivo* CreA occupancy at the promoter of ethanol metabolism genes did not reduce under the ethanol-inducing condition when AlcR occupancy was significantly increased (Fig. 8G). In fact, higher CreA occupancy levels were observed at these as well as many other CreA and AlcR cooccupied promoters (Fig. S7A). Systematic analysis showed a positive correlation between CreA and AlcR ChIP-seq signals among cooccupied promoters, regardless of whether the promoter has overlapping or distinct CreA and AlcR occupancy patterns (Fig. 8H).

10.1128/mbio.03734-21.7 FIG S7 DNA binding competition with pathway specific factors is not a universal mechanism for CreA repression. (A) Genome browser screenshots for CreA and AlcR ChIP-seq binding signals on the promoter of the representative genes under glucose and ethanol conditions. The upper display threshold set on the genome browser for each screenshot is indicated by the scale bar on the right. (B) Bar plots showing ChIP-qPCR results of CreA and AlcR binding levels at promoters not bound by AlcR in a double-tagged strain grown under repressing (glucose), inducing (ethanol), and induced-repressed (glucose and ethanol) carbon conditions. Download FIG S7, PDF file, 0.6 MB. Copyright © 2022 Chen et al. 2022 Chen et al. https://creativecommons.org/licenses/by/4.0/ This content is distributed under the terms of the Creative Commons Attribution 4.0 International license.

In light of this result and to rule out any potential technical artifact related to sequencing, an independent set of ChIP experiments against CreA^GFP^ and AlcR^Myc^ was carried out in a double-tagged strain grown under repressing (glucose), inducing (ethanol), and induced-repressed (glucose and ethanol) carbon conditions. Consistent with the above-described ChIP-seq result, increased AlcR^Myc^ occupancies were observed at ethanol metabolism genes in the presence of ethanol (e.g., ethanol or ethanol and glucose as the carbon source) (Fig. 8I). More importantly, CreA^GFP^ occupancy at these promoters was also elevated (Fig. 8I) but did not change at promoters not subjected to AlcR control (Fig. S7B). This is inconsistent with the expectation (a negative correlation) for the competition model, suggesting that direct DNA binding competition with transcriptional activators is not a universal mechanism for CreA repression. The positive correlation also suggests a potential role of CreA in the transcriptional activation process.

### CreA exerts global regulatory effects in a hierarchical manner.

**FIG 9:**
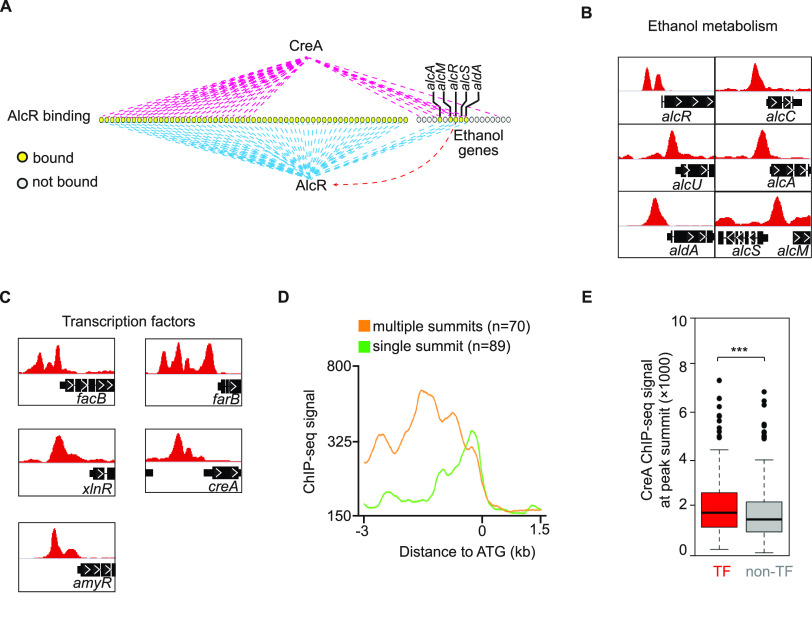
CreA exerts its global effects through controlling expression of transcription factor genes but does not compete with their DNA binding. (A) A schematic diagram showing common and unique CreA and AlcR gene targets. The genes for ethanol metabolism are indicated. (B and C) Genome browser screenshots for CreA binding signals on the regulatory (*alcR*) and structure genes (*alcC*, *alcU*, *alcA*, *aldA*, *alcS*, and *alcM*) for ethanol metabolism (B) and representative transcription factor genes (C). (D) A line plot showing CreA binding levels at the promoter of transcription factor genes with single or multiple binding peaks. (E) A boxplot showing overall CreA ChIP-seq signals on peak summits for TF and non-TF genes. Error bars represent standard deviations of signals. Average signal is indicated by the black line in the box. *t* test was used for statistical significance. ***, *P* ≤ 0.001.

For some pathways, CreA exerts its regulatory effect through a mechanism in which CreA directly represses both an upstream transcriptional regulator as well as the regulator’s downstream targets to ensure tight control (10, 19, 22). Interestingly, a comparison between AlcR and CreA targets revealed only about 50% (*n* = 33 out of 62) of AlcR target genes were also bound by CreA (Fig. 8C and 9A). In other words, there are many more AlcR targets not directly controlled by CreA. This result also indicates that CreA can expand its regulatory effects through AlcR functions.

Remarkably, the integrated analysis showed that more than 35% of DNA-binding transcription factor-encoding genes in the A. nidulans genome (216 out of 593) were under CreA control (Fig. S8). Of these, 159 had CreA occupancy at their promoters (Fig. S8), corresponding to more than 10% of the overall CreA direct regulon. Many established transcription factors controlling various physiological pathways and processes, including those enriched among the genes of class 1 to 4, were direct targets of CreA. For example, CreA bound to the promoter of the transcription factors regulating nitrogen metabolite repression (*areA*, *tamA*, *nmrA*, and *meaB*), metabolic pathways (e.g., *amyR* for starch metabolism, *xlnR* for xylan/xylose metabolism, *clrB* for cellulose metabolism, *facB* for acetate metabolism, *amdX* for acetamide metabolism, *farB* and *scfA* for fatty acid metabolism, *alcR* for ethanol metabolism, *rhaR* for rhamnose metabolism, *cpcA* for amino acid metabolism, *metR* and *sconB* for sulfur metabolism, and *apdR*, *dbaG*, and *mtfA* for secondary metabolism), development (e.g., *atfA*, *devR*, *flbC*, *flbD*, *nosA*, *nsdC*, *nsdD*, *sfgA*, *steA*, and *stuA*), siderophore biosynthesis (e.g., *sreA*), UPR (e.g., *hacA*), and responses to nutrient (e.g., *xprG*), pH (e.g., *pacC*), zinc limitations (*zapA*), and oxidative stress (e.g., *rsrA*) (Fig. S8). Considering transcription factors often control many downstream genes, this observation is consistent with and probably explains the substantial indirect effects observed in the *creA*Δ mutant.

10.1128/mbio.03734-21.8 FIG S8 CreA controls a large number of transcription factor genes. The table shows the list of CreA-controlled transcription factor genes (e.g., bound by CreA and/or expression significantly altered in *creAΔ*). Their gene expression changes in the *creA*Δ mutant compared to the wild type are indicated by blue and red cell colors for down- and upregulation, respectively. The genes bound by CreA are marked with a black line. Download FIG S8, PDF file, 0.9 MB. Copyright © 2022 Chen et al. 2022 Chen et al. https://creativecommons.org/licenses/by/4.0/ This content is distributed under the terms of the Creative Commons Attribution 4.0 International license.

We noted an interesting pattern in that there are two CreA ChIP-seq peaks at the *alcR* promoter but only one for all other ethanol metabolism genes (Fig. 9B). This observation potentially suggests a tighter regulation by CreA on the pathway-specific transcriptional regulator AlcR than for its downstream metabolism genes. The same trend (i.e., multiple CreA occupied sites) was observed at the promoters of *facB* and *xlnR*, *amyR*, and *farB*, the regulator genes for acetate, xylose/xylan, starch, and fatty acid metabolism (Fig. 9C), respectively, as well as 65 other transcriptional regulator genes (Data Set S13 at https://figshare.com/s/9f324a2370691f209579), including *creA* itself. The CreA occupancy levels at these regulatory gene promoters were generally higher than those at the promoter of nonregulatory genes (Fig. 9D and E), indicating tighter and more elaborate CreA control on upstream regulators than downstream genes. Taken together, our overall work identified the regulatory network of the master regulator CreA and revealed how CreA exerts its global regulatory effects on diverse physiological processes in a hierarchal manner.

## DISCUSSION

### Functional divergence between CreA and Mig1/Scr1.

**FIG 10:**
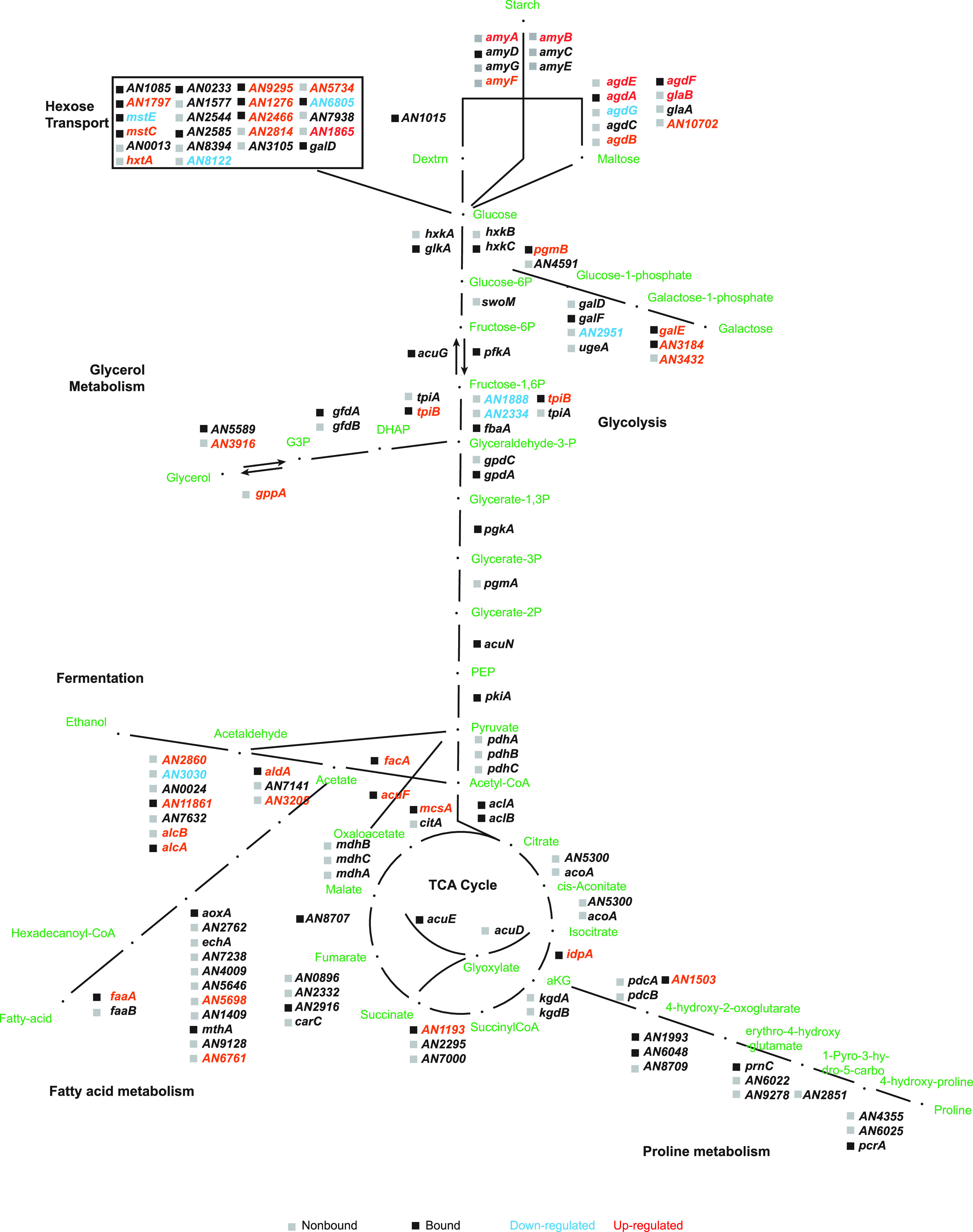
Overview of the direct and indirect roles of CreA on carbon metabolism pathways. A summary of CreA binding and transcriptional effect on the genes of various carbon metabolism pathways is shown. The presence or absence of CreA binding at the respective promoter is indicated by solid black and gray boxes, respectively. Up- and downregulated genes in the *creAΔ* mutant relative to the wild type are colored red and blue, respectively.

This study has applied an integrated analysis of RNA-seq and ChIP-seq data to delineate the direct and indirect actions of CreA and has not only confirmed the well-established role of CreA in carbon metabolism (Fig. 10) but also uncovered additional diverse cellular functions and physiological processes, including secondary metabolism, sexual and asexual development, iron homeostasis, and responses to oxidative and unfolded protein stresses. While these pleiotropic CreA functions are consistent with the severe growth phenotype observed in the *creA*Δ mutant, the finding is in stark contrast to S. cerevisiae Mig1 and S. pombe Scr1, which mainly control carbon metabolism (6, 63). Also distinct from Mig1 and Scr1 is the fact that CreA could bind to its targets regardless of carbon conditions. It is noteworthy that the number of CreA-bound genes is significantly larger than those of Mig1 and Scr1, and interestingly, many of the CreA targets are unique to filamentous fungi. These findings strongly suggest an interesting functional divergence between CreA and Mig1/Scr1, although it is currently unclear whether CreA has expanded functions in the filamentous fungal lineage or Mig1/Scr1 has lost functions in the degenerate yeasts. A similar suggestion was put forward for A. fumigatus by a recent study (47). However, in the other filamentous fungi T. reesei and N. crassa, the growth defects of the corresponding *creA* mutants are mainly observed on specific carbon sources (43, 68), implying that these CreA orthologues primarily serve a carbon-specific role, as with Mig1 and Scr1 in the two yeasts.

It is noteworthy that a significant fraction of CreA binding sites lack the conserved Mig1/CreA consensus motif SYGGRG but are enriched with two motifs carrying a core CGGG sequence. It remains to be determined whether these bindings are direct (involving its DNA binding domain) or indirect (through protein-protein interactions with a transcription factor that binds to the CGGG motif, e.g., as a transcription cofactor [69]). It is noteworthy that mutations disrupting the CreA DNA binding domain cause a less extreme growth phenotype than mutants lacking other parts of or the entire CreA coding region (28, 48, 49), indicating protein-protein interactions are important for the overall CreA functions.

### CreA couples the intracellular carbon status with development.

Genetic studies had previously implicated a role of CreA in asexual development (28). We have confirmed a conidiation phenotype in the *creA*Δ mutant and further observed a hypersexual phenotype that has not been reported previously. Our functional genomic data identified many upstream developmental regulators as direct targets of CreA. As carbon quality and availability affect CreA levels and, hence, function, our findings suggest a mechanistic link coupling cellular carbon status with developmental fate. This may be mediated through controlling the expression of developmental regulators (Fig. S7) and/or affecting nutrient sensing by G-protein-coupled receptors (70, 71). The coupling of carbon metabolism with development may ensure the success of the organism in the ever-changing environment, e.g., reproducing rapidly and clonally in the form of spores through asexual development under metabolic favorable environments for efficient dispersal, whereas under less nutritional conditions A. nidulans undergoes sexual development to produce and encapsulate highly stress-resistant ascospores (72) in a protective structure. We further suggest that CreA actually couples the developmental processes with other nutritional statuses in addition to carbon, as many transporters for nitrogen and amino acids are also targets of CreA. Therefore, this work indicates CreA is a bona fide global regulator in A. nidulans coordinating cellular metabolism and development.

### High intracellular glucose levels induce N-glycan biosynthesis and unfolded protein stress.

The observation that genes involved in ER function and unfolded protein response were induced along with increased *hacA* activation strongly indicates the *creA*Δ mutation results in unfolded protein stress. This and previous studies (73, 74) showed that the high-affinity glucose transport system is derepressed in the mutant, and presumably glucose influx would be increased significantly. It is noteworthy that the metabolic fluxes through the central carbon metabolism pathway are actually minimally altered in the *creA*Δ mutant compared to the wild type (75). A similar situation was also reported for A. fumigatus in which a rise in the intracellular glucose level was observed in the *creA*Δ mutant, but the levels of metabolites from the early steps of glycolysis (e.g., glucose-6-phoshate and fructose-6-phosphate) were not significantly changed (47). Therefore, the loss of CreA may cause a homeostatic imbalance between glucose uptake and glycolysis under situations when glucose supply in the environment is abundant. Interestingly, under our experimental setup (1% glucose as the sole carbon source), we found that N-glycan biosynthesis genes were also markedly upregulated in the mutant. As N-glycan is a polymer of sugars such as glucose and mannose (which can be converted from fructose-6-phosphate), the upregulation suggests a situation whereby the elevated intracellular glucose in the *creA*Δ mutant is diverting the metabolic flux to the biosynthesis of N-glycan that, in turn, affects protein processing and folding in the ER (76). However, the unfolded protein stress alone is probably not the cause of the severe mutant growth phenotype, as growth of the *creA*Δ mutant is similarly impaired when growing on media containing alternative carbon sources such as acetate and proline, which do not lead to intracellular glucose buildup and unfolded protein stress. Nevertheless, these overall findings provide a physiological connection between excessive intracellular glucose, N-glycan biosynthesis, ER function, and unfolded protein stress in A. nidulans.

### CreA is a master regulator controlling diverse pathways through a hierarchical regulatory network.

CreA is known to repress both upstream regulators (e.g., pathway specific activators) and their downstream target genes, a regulatory mechanism commonly known as a double-lock control (see reference 77 for a review). It was proposed that CreA physically competes with upstream regulators (e.g., AlcR) for DNA binding at the promoter of downstream genes to repress their expression (i.e., block transcriptional activation) (19). However, we found that CreA and pathway-specific activators occupy distinct nonoverlapping target sites at many promoters, and their occupancies are positively correlated (rather than anticorrelated, as expected for DNA binding competition), suggesting direct competition is not a universal mechanism for CreA repression.

Moreover, our genome-wide binding analysis of CreA and AlcR revealed that only a small number of genes within the ethanol metabolism pathway and AlcR direct targets are actually subjected to the double-lock control, while expression of the majority of downstream pathway genes is indirectly regulated by CreA as a result of the derepression of the transcription factors in the *creA*Δ mutant, consistent with an early suggestion for the regulation of xylanolytic genes by CreA and XlnR (40). This pyramid-shaped regulatory pattern conforms to what was defined as the hierarchical regulatory model (78–80). The extreme growth phenotype of the *creA*Δ mutant is consistent with the expectation for top-level transcriptional regulators of hierarchical networks (78–80). This work now places CreA at the top of the hierarchy for multiple different networks of genes involved in diverse physiological pathways and processes in addition to carbon metabolism. Therefore, CreA is a bona fide master regulator in A. nidulans.

Interestingly, we discovered that CreA occupies multiple locations at the promoter of many transcription factors. Clustering of transcription factor binding sites for the same transcription factor at promoters and enhancers (known as homotypic clusters) is a conserved *cis*-regulatory organization in vertebrate and invertebrate animals (81), while homotypic clustering is not common in S. cerevisiae (82). It has been found that homotypic clustering is enriched for transcription factor and developmental genes in both human and *Drosophila* (83, 84). Of note, multiple CreA bindings were also observed at developmental genes’ promoter in A. nidulans, suggesting that the organization of *cis*-regulatory elements at A. nidulans promoters (at least for transcription factor and developmental genes) more closely resembles the situations in higher eukaryotes than its fungal relative, S. cerevisiae.

Homotypic clusters are believed to facilitate transcriptional regulation through facilitating recruitment of transcription factors to targets, modulating transcription factor binding activity according to its cellular concentration, or promoting interactions with different regulatory complexes (81, 85). The presence of multiple binding sites also provides functional redundancy (86, 87) and thereby could serve as a backup in case of spontaneous mutation removing one of the cognate sites. Therefore, we propose that A. nidulans evolved multiple CreA binding sites to secure tight repression of important pleiotropic genes, such as those encoding transcription factors and developmental regulators, the misexpression of which could lead to adverse physiological and developmental consequences, as clearly seen from the phenotype of the *creA*Δ mutant. As such, CreA exerts different levels of security controls (e.g., homotypic clustering versus double lock) on the target genes at different levels of the hierarchy.

### How is CreA function at diverse promoters regulated?

In S. cerevisiae, it has been well established that Mig1 is controlled at the level of nuclear localization (88). Whether A. nidulans CreA is regulated at the level of subcellular localization is controversial. A number of studies have shown that the localization of the GFP-CreA fusion protein is regulated by carbon sources; for example, CreA was observed in the nucleus when cells are grown under CCR conditions, while it is relocated to the cytoplasm under non-CCR conditions (30, 32). However, CreA has been shown to undergo posttranslational cleavage (27, 29, 30), and it was noted in one of these studies that the GFP-CreA fusion is subjected to proteolytic degradation under the experimental conditions examined (30). When GFP was introduced to a region of CreA that is not affected by degradation (e.g., the C terminus), CreA was found to localize in the nucleus regardless of CCR conditions (29). Our ChIP-seq analysis also demonstrated constitutive CreA binding to DNA under different CCR conditions, including carbon starvation, which is likely the only non-CCR condition. Therefore, some level of CreA must exist in the nucleus, even under non-CCR conditions.

The constitutive occupancy of CreA at promoters under both CCR and non-CCR conditions also indicates posttranslational controls in modulating CreA-mediated repression. CreA is subject to differential phosphorylation under different CCR conditions (34). Phosphorylation controls the nuclear import kinetics and transcriptional repression activity of CreA (33). In S. cerevisiae, protein kinases (including Snf1, which phosphorylates the CreA orthologue Mig1 to relieve its repression) can be recruited to promoters by transcription factors for transcriptional activation (89–93). Kinases at promoters can phosphorylate and switch the Tup1-Ssn6 corepressor, which is recruited to promoters by Mig1 and other repressors, into a coactivator complex (94). In the absence of the Tup1-Ssn6 corepressing function, those repressor proteins can also act as activators to recruit additional coactivator complexes for transcriptional activation (94, 95). Although earlier studies showed that the Tup1 orthologue (RcoA) is not required for CCR in A. nidulans (96, 97), CreA can physically interact with RcoA and SsnF (31). The CreA orthologue Cre1 in T. reesei can also be converted from a transcriptional repressor to an activator (98), and direct positive roles of CreA are also found for a significant number of CreA-bound targets (class 4 genes) (Fig. 3A). More importantly, we observed a strong positive correlation between the genomic occupancy of CreA and the pathway-specific factor AlcR under AlcR-activating conditions, suggesting that the CreA-RcoA-SsnF complex is involved in the transcriptional activation process. Based on these, we propose a model in which pathway-specific activators recruit protein kinase(s) to promoters for phosphorylation of CreA to manipulate its regulatory properties in a promoter-specific manner. Phosphorylation of CreA by different kinases recruited by different transcriptional activators is expected to have distinct regulatory effects. Identifying the regulatory phosphorylation sites and the kinases involved will shed light on how the diverse functions of this master regulator are regulated.

## MATERIALS AND METHODS

### Strains and growth conditions.

Strains used in this study are listed in Data Set S14 at https://figshare.com/s/9f324a2370691f209579. The *creA99* mutant was isolated previously (28). Deletion and epitope-tagged strains were constructed using oligonucleotides listed in Data Set S14, as described previously (99). For both RNA-seq and ChIP-seq experiments, strains were cultured in Aspergillus nitrogen-free minimal (ANM) medium containing 1% glucose and 10 mM ammonium tartrate at 37°C for 16 h and then transferred to ANM medium containing 10 mM ammonium chloride with or without a carbon source (e.g., 1% glucose, 50 mM acetate, 50 mM proline, or 1% ethanol) for 6 h at 37°C with continuous shaking at 220 rpm. For the comparison of CreA^GFP^ binding under normal and overexpression conditions, the WT(p)*creA^GFP^* and *gpdA*(p)*creA^GFP^* strains were grown in ANM medium containing 1% glucose and 10 mM ammonium tartrate supplemented with 0.1% fructose and 0.05% arginine at 37°C for 24 h. For the growth assays, strains were point inoculated at the center of solid ANM medium containing the indicated carbon compounds as the sole carbon source or containing 1% glucose with milk, allyl alcohol, ferric iron, or hydrogen peroxide at the indicated concentrations and grown at 37°C for 3 days.

### RNA extraction and library preparation for RNA-seq.

Strains were grown as described above with 1% glucose as the sole carbon source, and mycelia were harvested through filtering using Miracloth (Millipore, Billerica MA, USA), rinsed with cold double-distilled water (ddH_2_O), and pressed-dried on paper towel. The dried mycelium pad was immediately frozen in liquid N_2_ and kept at –80°C until use. RNA was extracted with TRIzol (9109; TaKaRa) according to the manufacturer's protocol with a modification in the mycelium lysis step, in which mycelia were lysed in TRIzol with five rounds of 3 min beating using a Bullet Blender (Next Advance) with at least 3 min of cooling between cycles. Homogenized samples were incubated at 56°C for 10 min to dissociate the nucleoprotein complex completely before adding chloroform. RNA integrity was analyzed on an Agilent Bioanalyzer 2100 (Stockport, UK) using an Agilent RNA 6000 Nano kit (no. 9780). RNA libraries were prepared using a NEBNext Ultra directional RNA library prep kit (7760; NEB) as per the manufacturer’s instructions and sequenced on the Illumina HiSeq2500 platform at the Genomics and Single-Cell Analysis Core facility at the University of Macau.

### Chromatin preparation.

Untagged wild-type and CreA-tagged strains were grown under the conditions described above. Mycelia were cross-linked using formaldehyde at a final concentration of 1% with gentle shaking at room temperature for 20 min. To stop the cross-linking, 25 mL of 2.5 M glycine was added to the culture and mixed with gentle shaking at room temperature for 10 min. Mycelia were harvested through filtering using Miracloth (Millipore, Billerica MA, USA), rinsed with cold ddH_2_O, and pressed-dried on paper towel. The dried mycelium pad was immediately frozen in liquid N_2_ and kept at –80°C until use. Chromatin preparation was carried out as described previously (100), with minor modifications for mycelium samples. For each sample, around 25 mg of mycelia was lyophilized for 2 h and lysed in 500 μL of FA lysis buffer (150 mM NaCl) by six cycles of lysis, each for 3 min, using a Bullet Blender (Next Advance) with at least 3 min of cooling between cycles. Chromatin was pelleted by centrifugation at 14,000 rpm for 15 min at 4°C and then resuspended in 500 μL of FA lysis buffer (150 mM NaCl). Sonication was used to shear chromatin DNA to around 100 to 500 bp. Chromatin solution was recovered by centrifugation at 14,000 rpm for 30 min at 4°C and kept at –80°C until use.

### Chromatin immunoprecipitation.

Immunoprecipitation was performed as described previously (95) using antibodies against HA (sc-7392; F7; Santa Cruz), MYC (sc-40; 9E10; Santa Cruz), FLAG (F1804; M2; Sigma), or GFP (ab290; Abcam). Briefly, 2 to 4 μg of chromatin extract was mixed with 2 μg of antibody in an end-to-end rotator for 1.5 h at room temperature, followed by adding ∼15 μL packed protein A-Sepharose (GE17-0780-01; GE Healthcare) to the mixture and rotated for another 1.5 h. Subsequently, protein A-Sepharose matrix was transferred to a Corning Costar SpinX centrifuge tube filter and washed twice with FA lysis buffer (150 mM NaCl), once with FA lysis buffer (500 mM NaCl), once with ChIP wash buffer, and once with Tris-EDTA buffer, as described previously (95, 100). Immunoprecipitated chromatin DNA was de-cross-linked at 65°C overnight and purified using a Qiagen PCR clean-up purification kit (28106; Qiagen).

### ChIP-qPCR.

Real-time PCR was carried out on purified immunoprecipitated DNA and input samples using primers listed in Data Set S14.

### Library preparation for Illumina sequencing.

A small fraction of purified IP product was used to confirm positive ChIP signal by real-time PCR before subjecting the remaining sample to library preparation. Library preparation was performed as described previously (101), except the end repair was carried out using the NEBNext Ultra II end repair/dA-tailing module (E7546L; NEB) according to the manufacturer's protocol. For input controls, 1 ng of chromatin DNA was used for library preparation. Libraries were checked and quantified using a DNA high-sensitivity bioanalyzer assay (XF06BK50; Agilent), mixed at an equal molar ratio, and sequenced using the Illumina HiSeq2500 platform at the Genomics and Single-Cell Analysis Core facility at the University of Macau.

### Bioinformatics analysis.

For RNA-seq analysis, data for three independent biological experiments were obtained for each strain. Raw pair-end reads were aligned to Aspergillus nidulans FSGC genome assembly s10-m04-r03 using TopHat2 v2.1.1 with the following configurations: “–max-intron-length 1000 –min-intron-length 10 -p 8.” For differential gene expression analysis, normalized FPKM (fragments per million mapped reads) values for each annotated protein-coding gene were calculated by DEseq2 (v:1.22.2). Genes with a difference of 2-fold or more in expression were classified as DEGs. Correlation analysis showed that gene expression values between biological repeats for wild-type and *creA*Δ strains were well correlated (average *R* = 0.92 and 0.82, respectively) (see Fig. S9A in the supplemental material), while the correlation between the data of the wild type and the *creA*Δ mutant was significantly less (average *R* = 0.67; *P* < 10^−3^). Principal-component analysis (PCA) also showed good reproducibility between biological repeats and clear differences between the gene expression profiles of wild-type and *creA*Δ strains (Fig. S9B). Genes with very low or no expression (FPKM of <1 in at least two out of three biological repeats) in both wild-type and mutant strains or with inconsistent fold change between the wild type and mutant among the three biological repeats were excluded from downstream analysis (Data Set S1 at https://figshare.com/s/9f324a2370691f209579). Differentially expressed genes between the wild type and the *creA*Δ mutant were identified by DESeq2 (102), and genes with log_2_ fold change of  ≥1 or log_2_ fold change of ≤−1 and adjusted *P* values of ≤0.05 were classified as up- or downregulated genes, respectively. We also manually inspected the data and included genes showing consistent differential expression trends among all three biological repeats but were filtered out by *P* values due to variable fold changes among repeats. Figures for heatmap, PCA, correlation analysis, and other custom-made figures were plotted using in-house R scripts (R Core Team 2016). For ChIP-seq analysis, raw single-end reads were aligned to the Aspergillus nidulans FSGC genome assembly s10-m04-r03 using Bowtie2 (103) with the following configurations: “-5 5 -3 2 -q -p 2 –local.” Reads mapping to multiple genomic locations (i.e., ambiguous) were filtered out using SAMtools (104) with the following specifications: “samtools view -bq 1 -S.” To visualize genome-wide ChIP-seq binding on a genome browser, the data in .bam format were converted to .bdg format using MACS2 (105) with the parameters “macs2 pileup –extsize 200 -i,” and then the counts was normalized to FPKM value with an in-house bash script. ChIP-seq peaks were identified using MACS2 with the parameters “–nomodel -f BAM -g 30517219 –extsize 200 –m 5 500 -p 1e^−3^ –call-summits.” Only peaks with a distance between summits identified from two independent biological experiments within 100 bp for the same gene were included in the subsequent analysis. We also manually checked the MACS2-called peaks with marginal *P* values near the cutoff threshold on the Integrative Genomics Viewer (IGV) to exclude and include false positives and false negatives, respectively. The final lists of CreA binding summits for the CreA^HA^ and CreA^GFP^ strain are provided in Data Set S3 at https://figshare.com/s/9f324a2370691f209579. Correlation of ChIP-seq signals also showed a lower similarity between ChIP-seq experiments of the two different epitope-tagged CreA proteins (*R* = 0.59 for CreA^HA^ versus CreA^GFP^) (Fig. S10A) than between biological repeats (*R* = 0.90 for CreA^HA^ repeats, *R* = 0.89 for CreA^GFP^ repeats) (Fig. S10B). Upon close inspection of the CreA^HA^ and CreA^GFP^ unique binding sites, we found that both epitope-tagged CreA proteins were actually binding to an almost identical set of targets, just at slightly different levels, with some sites having signals below the filtering threshold of the MACS2 program (Fig. S10C and D). The minor differences in the binding patterns are probably due to subtle variations in ChIP efficiency with the different epitopes and/or antibodies. Therefore, the two lists of summits from the two tagged strains were combined for subsequent analysis. Summits were mapped to the annotation of the Aspergillus nidulans FSGC genome, version s10-m04-r03, excluding tRNAs and rRNAs using an in-house R-script package. The resultant gene lists were manually checked and confirmed on the IGV genome browser, especially for CreA bindings on multiple locations and at bidirectional gene promoters. *De novo* motif analysis was carried out on sequences (100 bp) spanning the summit of the CreA ChIP-seq peaks using MEME-ChIP (106). CreA motif 5′-SYGGRG-3′ was counted over a 100-bp window spanning each CreA summit. Density plots for ChIP-seq on the genome regions were calculated by counting the number of sequencing read fragments that overlapped within a given sliding window across the analyzed genome regions and then normalized to the total number of mapped reads in the respective data set and expressed as FPKM. Binding density plots across certain gene regions were plotted by R basic function image. Kyoto Encyclopedia of Genes and Genomes (KEGG) pathway annotation analysis was performed on the KEGG mapper website (https://www.genome.jp/kegg/tool/map_pathway1.html; release 95.2, 1 September 2020) (107). The gene list for the specific biological process/pathway used in the analysis was curated mainly based on the gene description and GO terms from the Aspergillus Genome Database (AspGD) (108, 109), FungiDB (fungidb.org) (110), KEGG, and literature references.

10.1128/mbio.03734-21.9 FIG S9 RNA-seq data quality. (A) Gene expression correlations between biological replicates of the wild type and the *creAΔ* mutant. The R value for each pairwise comparison was labeled on the top left. (B) PCA for RNA-seq data of the wild-type (in red) and *creAΔ* (in blue) strains. Two major components (PC1 and PC2) are displayed. Download FIG S9, PDF file, 1.6 MB. Copyright © 2022 Chen et al. 2022 Chen et al. https://creativecommons.org/licenses/by/4.0/ This content is distributed under the terms of the Creative Commons Attribution 4.0 International license.

10.1128/mbio.03734-21.10 FIG S10 ChIP-seq data quality. (A and B) Correlation analyses of ChIP-seq binding signals between (A) CreA^HA^ versus CreA^GFP^ and (B) biological repeats of CreA^HA^ and CreA^GFP^ ChIP-seq experiments. The R value for each correlation is presented. (C) Heatmaps displaying CreA^HA^ and CreA^GFP^ ChIP-seq and background (WT) signals over CreA binding sites identified in the CreA^HA^ and CreA^GFP^ strains. The signals across a 3-kb window spanning each CreA binding site are displayed. (D) Genome browser screenshots showing ChIP-seq signals at selected genes in the CreA^HA^, CreA^GFP^, and untagged wild-type control strains. The upper display threshold set on the genome browser for each screenshot is indicated by the scale bar on the right. Download FIG S10, PDF file, 2.7 MB. Copyright © 2022 Chen et al. 2022 Chen et al. https://creativecommons.org/licenses/by/4.0/ This content is distributed under the terms of the Creative Commons Attribution 4.0 International license.

### Total protein extraction and Western blot analysis.

Total protein extraction was performed as described previously (111), except for the lysis step. Frozen mycelia were lysed in 500 μl ice-cold denaturing buffer (10 mM Tris-HCl, pH 8.0, 25 mM NH_4_ acetate, 1 mM EDTA, 10% trichloroacetic acid) with an ∼100-μl volume of silica beads through six cycles of 3 min of beating using a Bullet Blender. Samples were allowed to cool on ice for at least 3 min between each cycle. Total protein concentration was measured using the Bio-Rad DC protein assay kit (5000111). The same amounts of proteins (50 μg) from different samples were subjected to SDS-PAGE and Western blotting. The anti-HA (Santa Cruz sc-7392 and Immunoway YM3003; 1:2,000 dilution) and anti-histone H3 (ab1791; 1:5,000 dilution; Abcam) primary antibodies and the horseradish peroxidase (HRP)-conjugated anti-rabbit (AP132P; 1:5,000 dilution; Merck Millipore) and HRP-conjugated anti-mouse (AP124P; 1:5,000 dilution; Merck Millipore) secondary antibodies were used.

### Asexual development phenotype characterization.

For the asexual phenotype analysis, wild-type and *creAΔ* mutant strains were point inoculated on solid complete medium and grown at 37°C for 3 days. Spores were then harvested by scraping and vortexed in 10 mL 0.01% Tween 80 and then filtered through a 40-*μ*m nylon mesh sterile filter (22-363-547; Fisherbrand) to separate spores from mycelial mass and debris. Each spore solution was diluted 500× in three separate tubes, the number of spores was counted using a hemocytometer, and the numbers from three counts were averaged and taken as one biological repeat. Three independent biological repeats were performed for each strain, and the averages are presented.

### Sexual development phenotype characterization.

For the sexual phenotype analysis, wild-type and *creAΔ* mutant strains were point inoculated on solid complete medium supplemented with 66 μM riboflavin and grown in the dark for 10 days at 37°C. After incubation, cleistothecia were harvested by scraping in 10 mL 0.01% Tween 80 and were collected using low-speed centrifugation (e.g., 1,000 rpm) at 4°C for 5 min. Subsequently, cleistothecia were washed twice with 10 mL 0.01% Tween 80. Three aliquots of 200 μl of sample were counted under the microscope, and the numbers from three counts were averaged and taken as one biological repeat. Three independent biological repeats were performed for each strain, and the averages are presented.

### Data availability.

Next-generation sequencing data are available from the NCBI SRA database under the accession numbers PRJNA689693, PRJNA689360, and PRJNA689345 for CreA, AlcR, and AcuK and AcuM ChIP-seq data, respectively, and PRJNA689667 for RNA-seq data. The R scripts for data analysis can be found on GitHub (https://github.com/donglg1309/CreA_Manuscript_2021). All supporting data sets are available at https://figshare.com/s/9f324a2370691f209579.
