# Supplementary figures and images for: Carbon Catabolite Repression Governs Diverse Physiological Processes and Development in Aspergillus nidulans
Source: mBio. 2022 Feb 15;13(1):e03734-21. doi: 10.1128/mbio.03734-21 (PMC8844935; doi:10.1128/mbio.03734-21)

**A**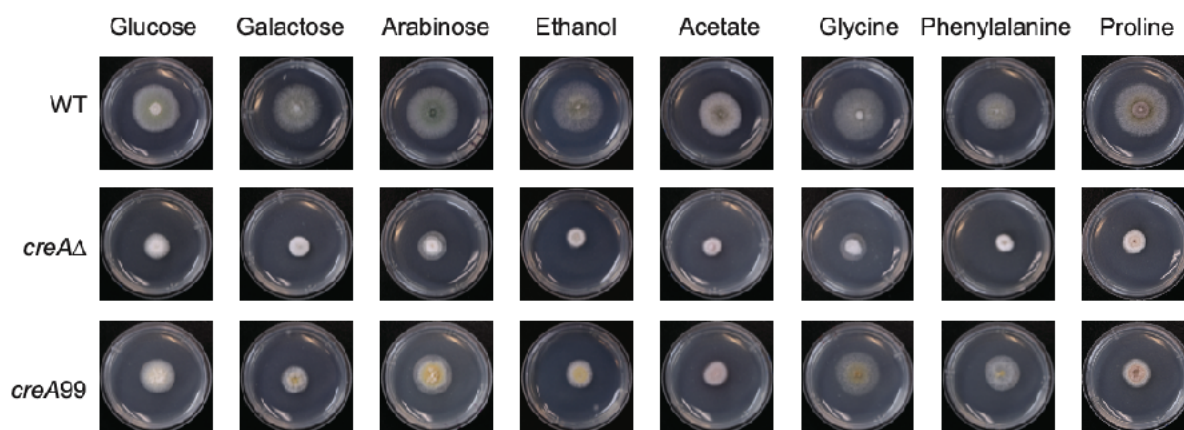**B**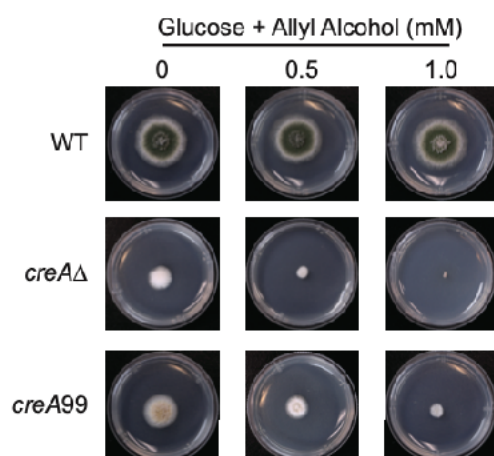**C**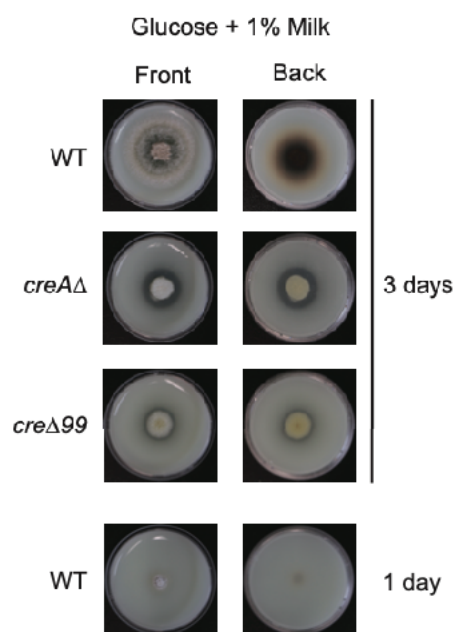

Supplement: FIG S1 [file mbio.03734-21-sf001.pdf]

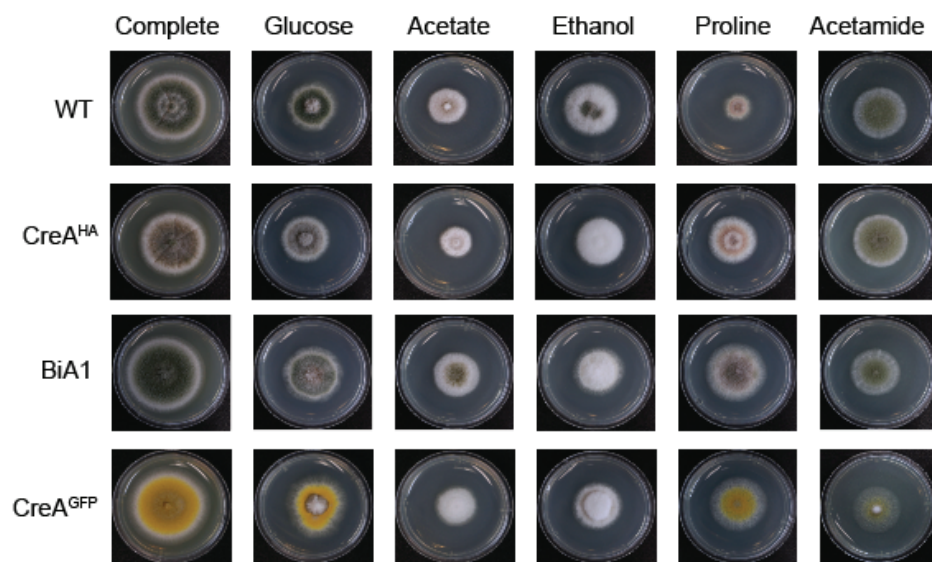

Supplement: FIG S2 [file mbio.03734-21-sf002.pdf]

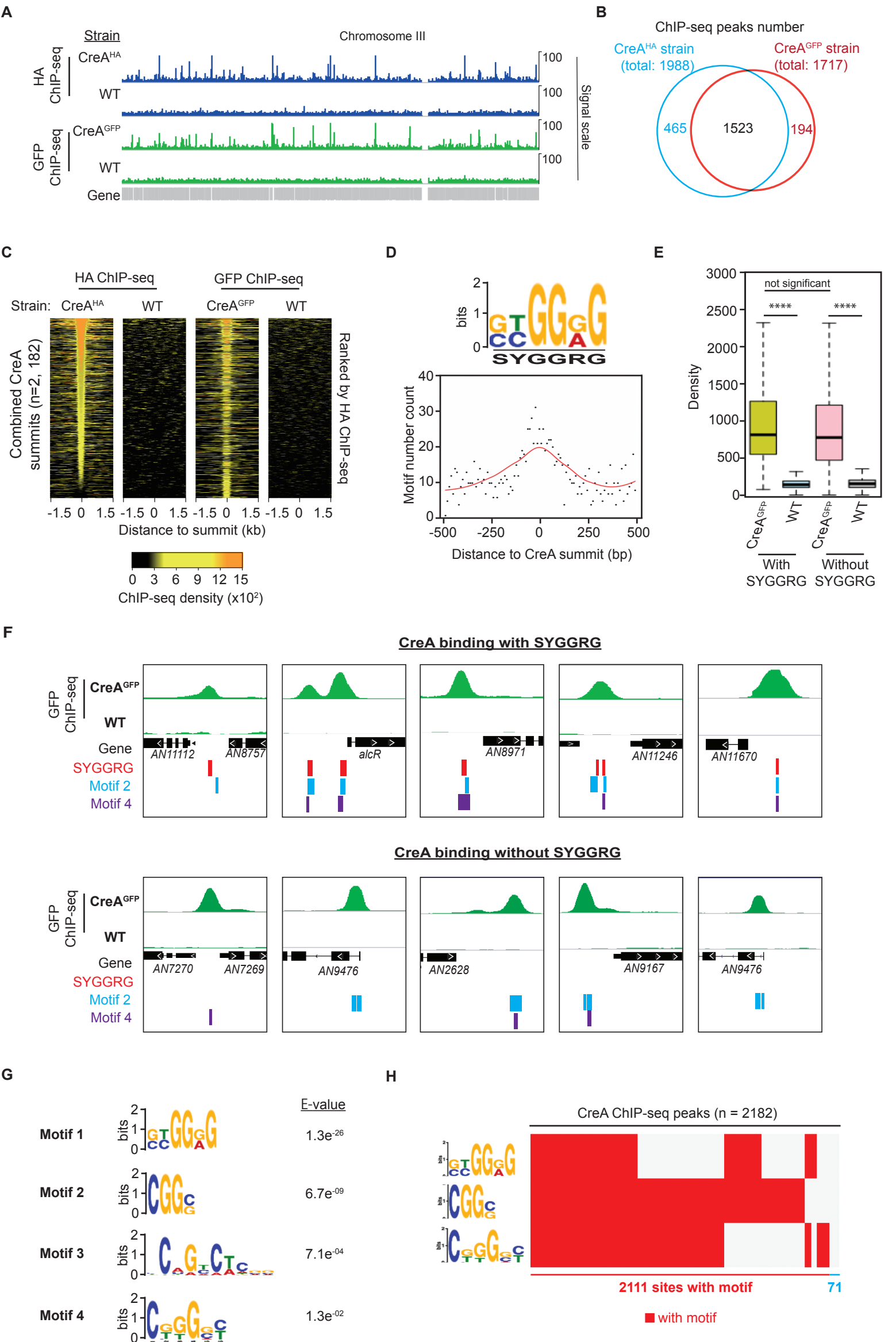

Supplement: FIG S3 [file mbio.03734-21-sf003.pdf]

**A**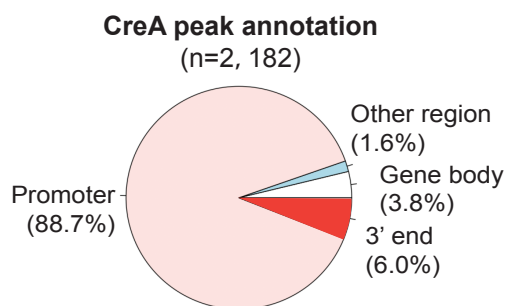**B**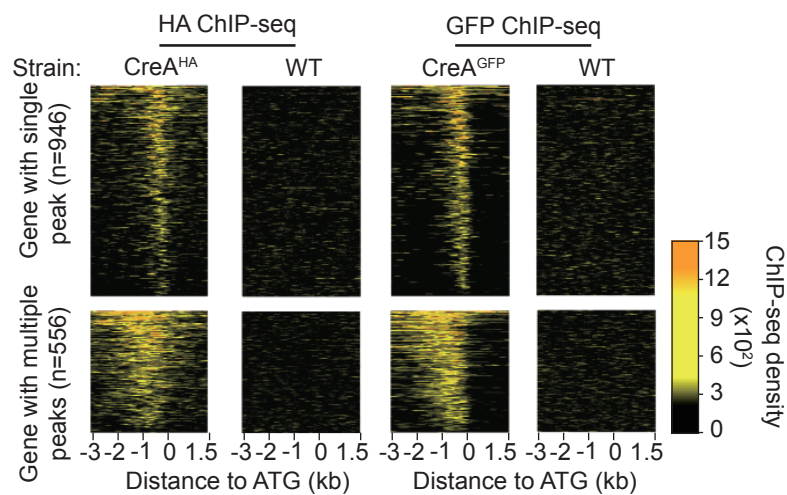**C**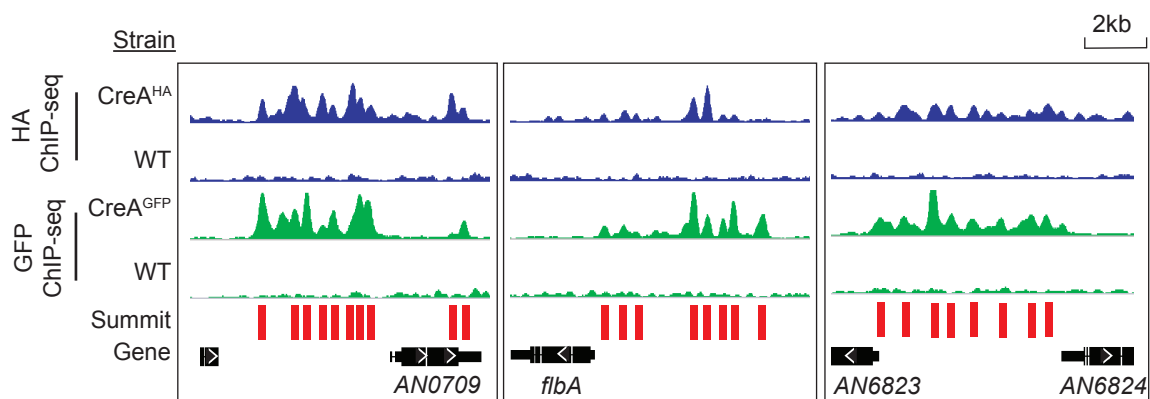

Supplement: FIG S4 [file mbio.03734-21-sf004.pdf]

**A**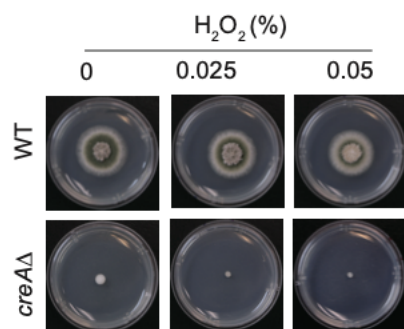**B**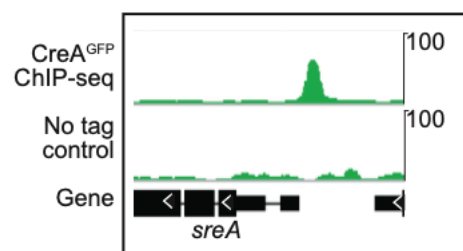**C**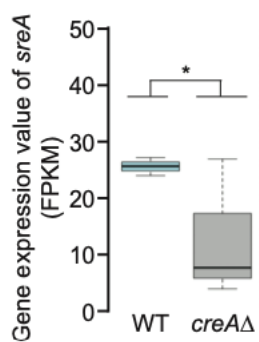**D**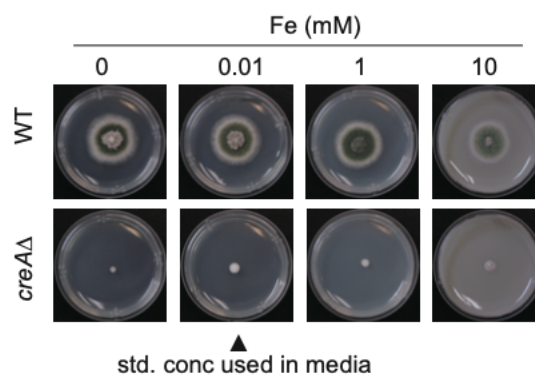**E**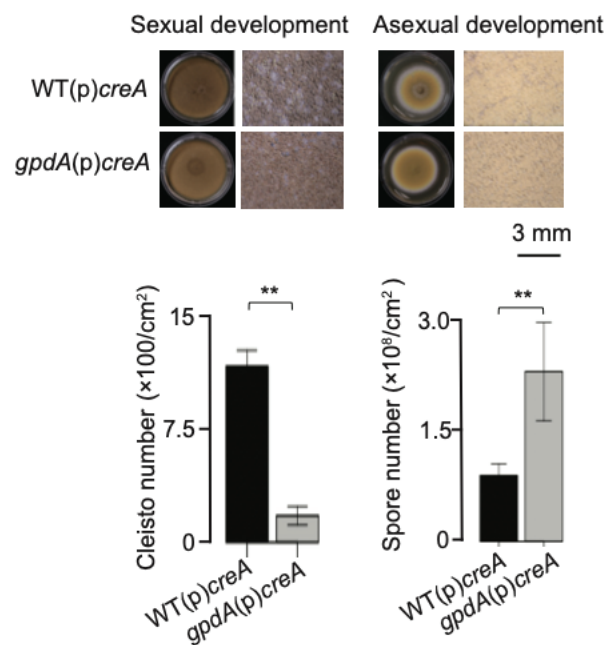

Supplement: FIG S5 [file mbio.03734-21-sf005.pdf]

**A**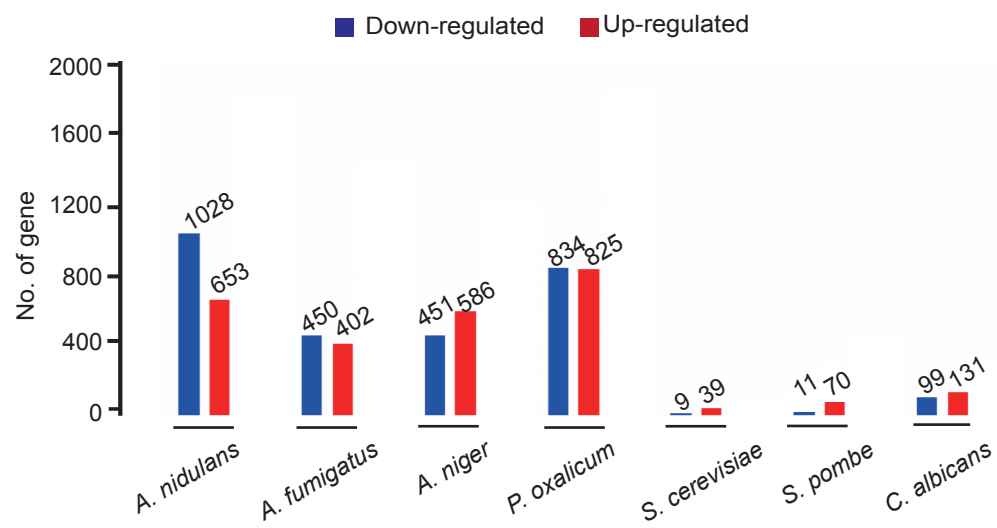**B**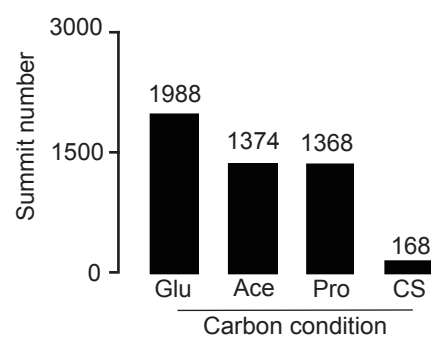

Supplement: FIG S6 [file mbio.03734-21-sf006.pdf]

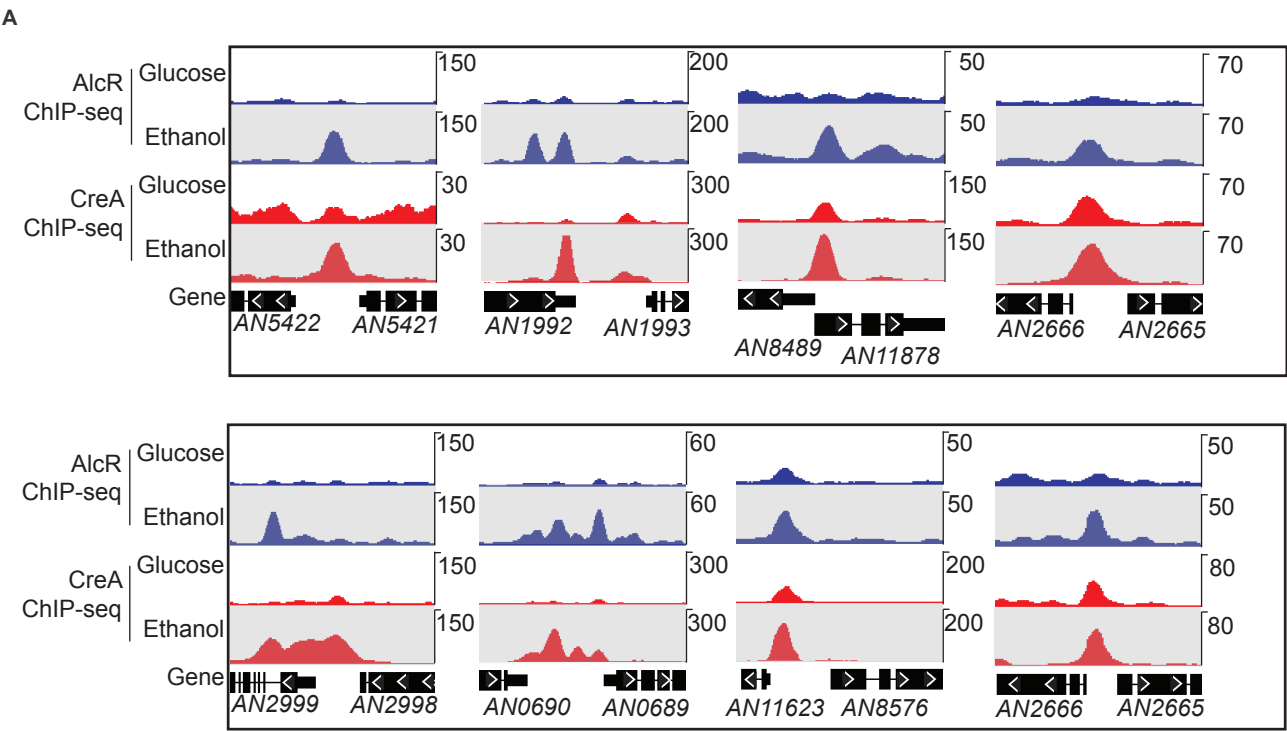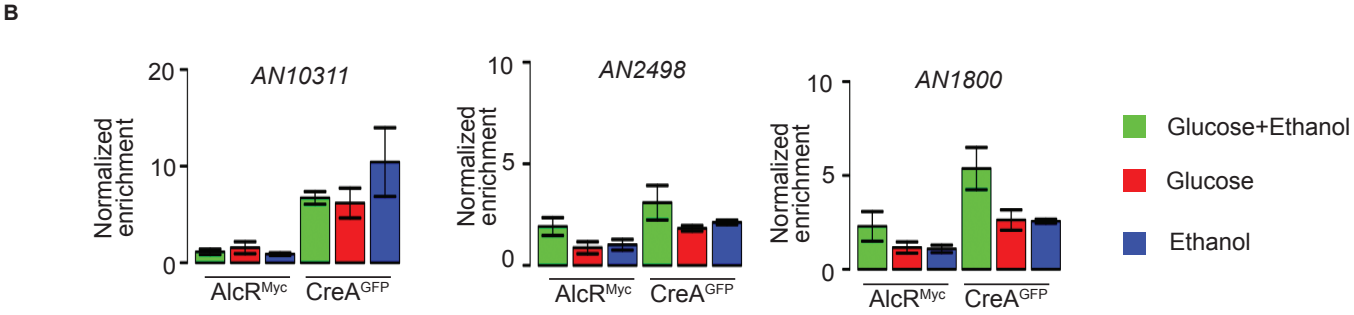

Supplement: FIG S7 [file mbio.03734-21-sf007.pdf]

**A**

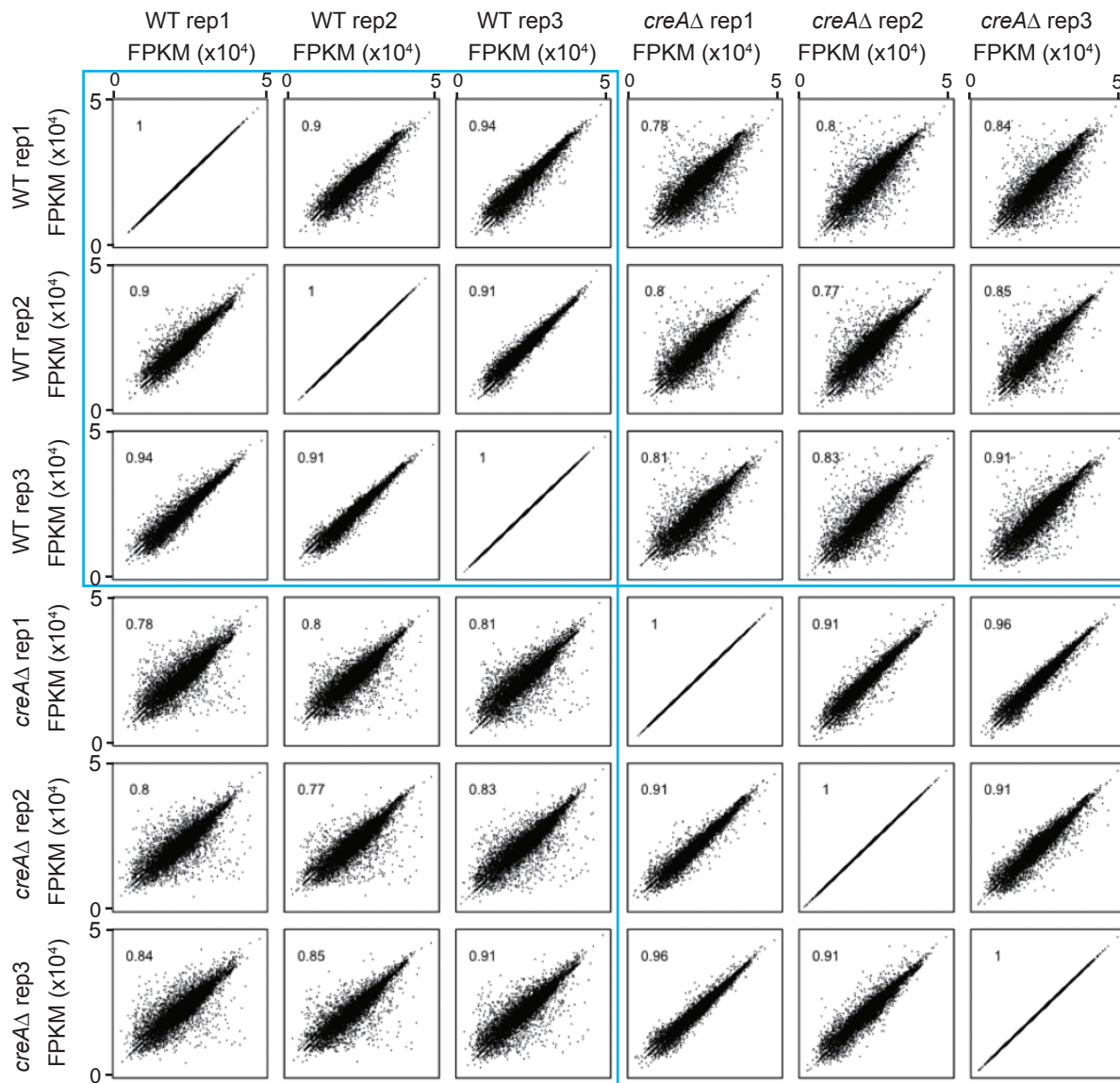

**B**

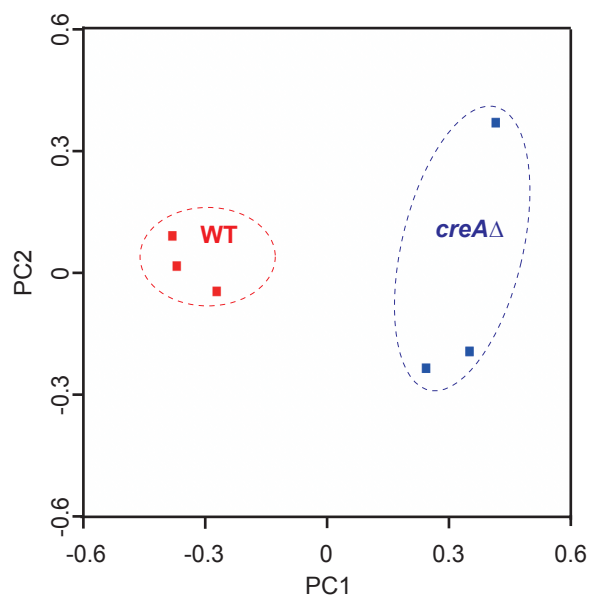

Supplement: FIG S9 [file mbio.03734-21-sf009.pdf]

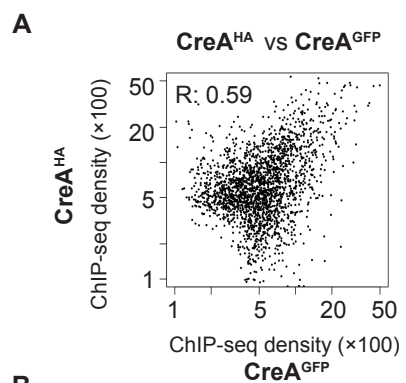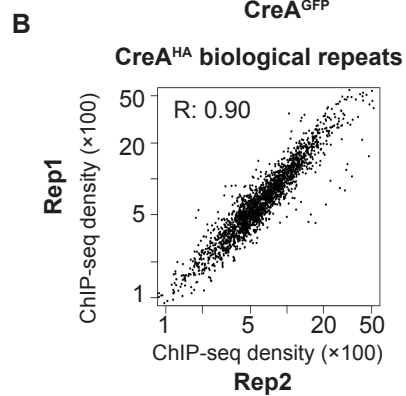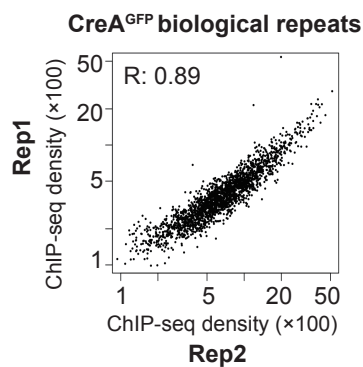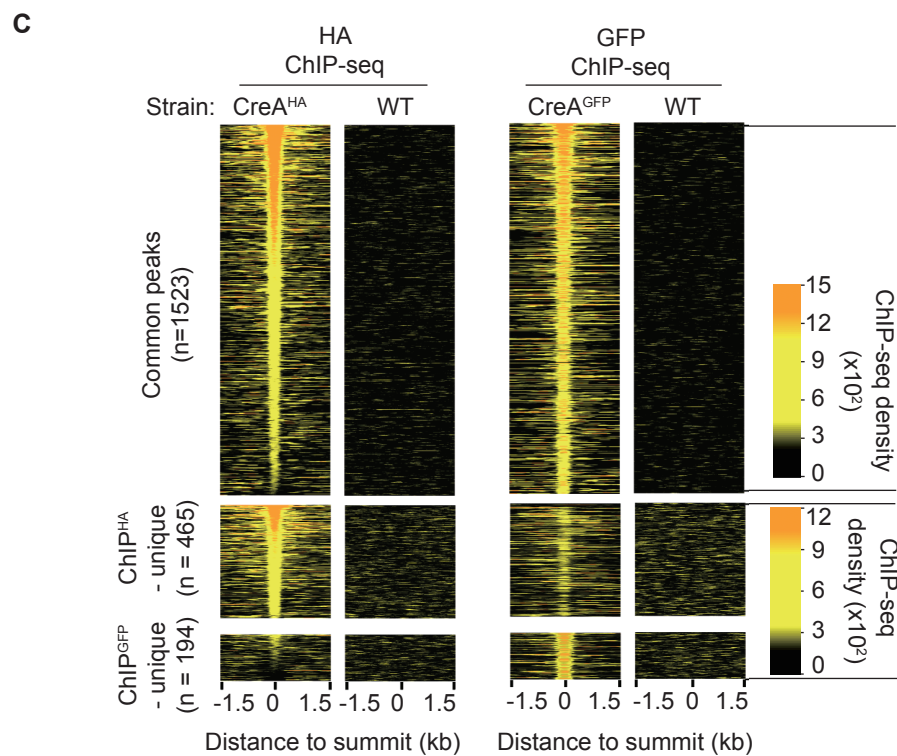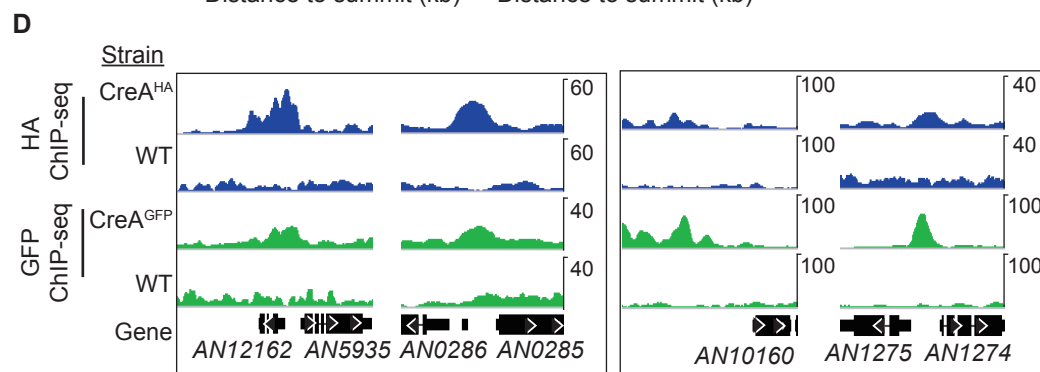

Supplement: FIG S10 [file mbio.03734-21-sf010.pdf]
