# Supplementary material for: Carbon Catabolite Repression Governs Diverse Physiological Processes and Development in Aspergillus nidulans
Source: mBio. 2022 Feb 15;13(1):e03734-21. doi: 10.1128/mbio.03734-21 (PMC8844935; doi:10.1128/mbio.03734-21)

Transcription factors controlled by CreA

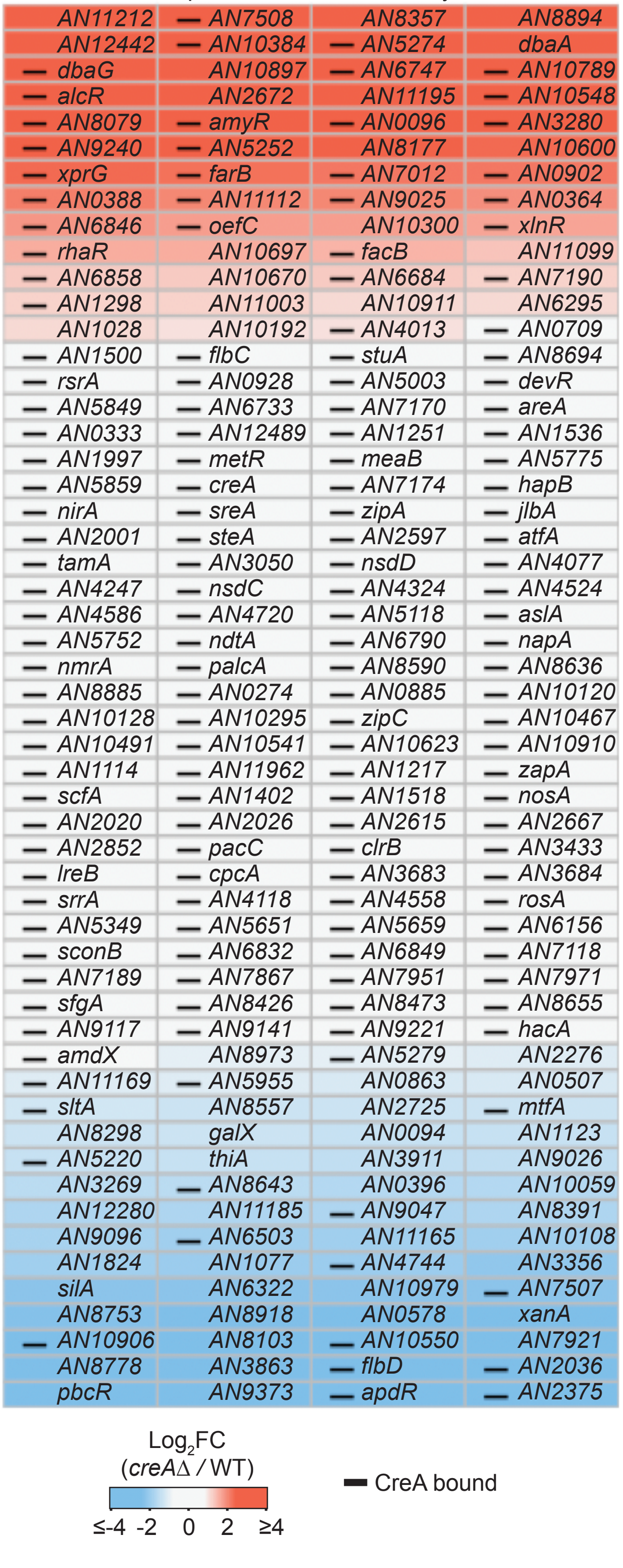

Supplement: FIG S8 [file mbio.03734-21-sf008.pdf]
